# Regulation of sarcomere formation and function in the healthy heart requires a titin intronic enhancer

**DOI:** 10.1172/JCI183353

**Published:** 2024-12-17

**Authors:** Yuri Kim, Seong Won Kim, David Saul, Meraj Neyazi, Manuel Schmid, Hiroko Wakimoto, Neil Slaven, Joshua H. Lee, Olivia Layton, Lauren K. Wasson, Justin H. Letendre, Feng Xiao, Jourdan K. Ewoldt, Konstantinos Gkatzis, Peter Sommer, Bénédicte Gobert, Nicolas Wiest-Daesslé, Quentin McAfee, Nandita Singhal, Mingyue Lun, Joshua M. Gorham, Zolt Arany, Arun Sharma, Christopher N. Toepfer, Gavin Y. Oudit, William T. Pu, Diane E. Dickel, Len A. Pennacchio, Axel Visel, Christopher S. Chen, J.G. Seidman, Christine E. Seidman

**Affiliations:** 1 Department of Genetics, Harvard Medical School, Boston, Massachusetts, USA.; 2 Division of Cardiovascular Medicine, Brigham and Women’s Hospital, Boston, Massachusetts, USA.; 3 Department of Cardiology, University Heart and Vascular Center Hamburg, University Medical Center Hamburg-Eppendorf, Hamburg, Germany.; 4 German Heart Center, Technical University of Munich, Munich, Germany.; 5 Environmental Genomics and Systems Biology Division, Lawrence Berkeley National Laboratory, Berkeley, California, USA.; 6 Department of Biomedical Engineering, Boston University, Boston, Massachusetts, USA.; 7 Department of Cardiology, Boston Children’s Hospital, Harvard Medical School, Boston, Massachusetts, USA.; 8 Ksilink, Strasbourg, France.; 9 Cardiovascular Institute, Department of Medicine, Perelman School of Medicine, University of Pennsylvania, Philadelphia, Pennsylvania, USA.; 10 Smidt Heart Institute, Cedars-Sinai Medical Center, Los Angeles, California, USA.; 11 Division of Cardiovascular Medicine, Radcliffe Department of Medicine and; 12 Wellcome Centre for Human Genetics, University of Oxford, Oxford, United Kingdom.; 13 Department of Medicine, University of Alberta, Edmonton, Alberta, Canada.; 14 Mazankowski Alberta Heart Institute, Edmonton, Alberta, Canada.; 15 Harvard Stem Cell Institute, Cambridge, Pennsylvania, USA.; 16 US Department of Energy Joint Genome Institute, One Cyclotron Road, Berkeley, California, USA.; 17 Comparative Biochemistry Program, UCB, Berkeley, California, USA.; 18 School of Natural Sciences, UCM, Merced, California, USA.; 19 Howard Hughes Medical Institute, Chevy Chase, Maryland, USA.

**Keywords:** Cardiology, Genetics, Cardiovascular disease, Genetic diseases, Heart failure

## Abstract

Heterozygous truncating variants in the sarcomere protein titin (TTN) are the most common genetic cause of heart failure. To understand mechanisms that regulate abundant cardiomyocyte (CM) *TTN* expression, we characterized highly conserved intron 1 sequences that exhibited dynamic changes in chromatin accessibility during differentiation of human CMs from induced pluripotent stem cells (hiPSC-CMs). Homozygous deletion of these sequences in mice caused embryonic lethality, whereas heterozygous mice showed an allele-specific reduction in *Ttn* expression. A 296 bp fragment of this element, denoted E1, was sufficient to drive expression of a reporter gene in hiPSC-CMs. Deletion of E1 downregulated *TTN* expression, impaired sarcomerogenesis, and decreased contractility in hiPSC-CMs. Site-directed mutagenesis of predicted binding sites of NK2 homeobox 5 (NKX2-5) and myocyte enhancer factor 2 (MEF2) within E1 abolished its transcriptional activity. In embryonic mice expressing E1 reporter gene constructs, we validated in vivo cardiac-specific activity of E1 and the requirement for NKX2-5– and MEF2-binding sequences. Moreover, isogenic hiPSC-CMs containing a rare E1 variant in the predicted MEF2-binding motif that was identified in a patient with unexplained dilated cardiomyopathy (DCM) showed reduced TTN expression. Together, these discoveries define an essential, functional enhancer that regulates *TTN* expression. Manipulation of this element may advance therapeutic strategies to treat DCM caused by *TTN* haploinsufficiency.

## Abstract 2

We identified a transcriptional enhancer of the essential sarcomere gene titin (TTN), which plays a critical role in TTN expression, sarcomere formation, and cardiomyocyte contractility.

## Introduction

Dilated cardiomyopathy (DCM) is characterized by left or biventricular dilatation with associated contractile dysfunction and occurs in approximately 1 in 250 individuals (1). Despite this considerable prevalence in the population, available treatment options are insufficient to slow or prevent the progression of DCM to heart failure, a devastating disorder associated with high morbidity and early mortality, including death for 25% of patients within 5 years of the first heart failure hospitalization (2).

DCM emerges in the setting of underlying cardiovascular or systemic diseases and as a primary myocardial disorder caused by damaging gene variants (3). Human genetic studies demonstrate that heterozygous truncating variants in the gene encoding titin (TTNtv) are the most common genetic cause of DCM. TTN is a highly expressed and essential protein in sarcomeres (4), the contractile unit in all striated muscles. TTN enables sarcomere assembly, contributes to mechano-chemical signaling, and regulates the passive stiffness of the sarcomere (5). Consisting of 34,350 amino acids, TTN is the largest human protein and contains 4 functionally distinct domains: the N-terminus anchors TTN at the sarcomere Z-disc, the I-band with repeating Ig-like domains modulates elastic properties of the TTN protein (6), the A-band interacts with other thick filament proteins, and the carboxyl M-band encodes a pseudokinase scaffold with mechanosensing and signaling properties (7). The functions of these domains are influenced by alternative exon splicing, particularly in the I-band (8). Pathogenic TTNtv in patients with DCM reside in exons (9, 10).

In addition to causing DCM, asymptomatic carriers of TTNtv with normal cardiac imaging show a subtle reduction in functional and morphologic heart parameters, including lower mean ejection fraction and left ventricular wall thickness compared with age-matched controls (10–12). TTNtv carriers also have increased susceptibility to DCM that arises in the context of pregnancy (13, 14), excessive alcohol consumption (15), and cancer therapies (16) but also have greater beneficial effects of acute ventricular unloading in comparison with other DCM genotypes (17–19). Together, these data indicate that TTNtv affect baseline cardiac structure and function and also impair the heart’s stress-adaptive mechanisms.

Emerging experimental data provide insights into the mechanisms underlying these clinical observations. Patient-derived and engineered human induced pluripotent stem cell–derived cardiomyocytes (hiPSC-CMs) with heterozygous *TTNtv* (*TTNtv/+*) have sarcomeres that are disorganized and less able to generate basal or stimulated force when constructed into cardiac microtissues than do WT hiPSC-CMs (20). *TTNtv/+* hiPSC-CMs also show a reduced capacity to reassemble sarcomeres after physical stress (21), and *Ttntv/+* mice have more protracted cardiac dysfunction than do WT mice after chemotherapy (16). We interpret these studies to indicate that appropriate stress responses by CMs necessitate increased production and incorporation of full-length TTN protein to support sarcomere function. This compensatory mechanism is attenuated in TTNtv carriers, which incites decompensation and unmasks genetic DCM.

We and others have reported the presence of truncated TTN protein in human DCM tissues (22, 23) and *TTNtv/+* hiPSC-CMs (20, 24), suggesting a potential contribution of a poison-peptide mechanism in addition to TTN haploinsufficiency in the development of DCM. Moreover, augmentation of WT TTN protein levels even in the presence of truncated TTN protein improves the contractility of hiPSC-CMs (25), probably because of improved sarcomere structure and biomechanical signaling from full-length TTN proteins. This hypothesis infers that understanding the regulatory control of *TTN* expression will inform mechanisms behind sarcomerogenesis in the healthy heart and in response to myocardial stress. Harnessing these mechanisms to increase the amount of TTN protein can serve as a treatment strategy for DCM caused by TTNtv (26). To our knowledge, however, neither the molecules nor sequences that promote *TTN* transcription are currently known.

Transcriptional enhancers and promoters are genetic element targets of tissue-specific transcription factors (TFs), which control spatiotemporal gene expression (27, 28). Recent studies have identified cardiac enhancers in developing mammalian hearts using computational analysis, chromosome conformation capture techniques (Hi-C), ChIP-Seq, and in vivo transgenic enhancer assays with zebrafish and mice (28, 29). Moreover, mice with biallelic deletion of computationally defined cardiac-specific enhancers for the cardiac β myosin heavy-chain (*Myh7*) and myosin light-chain (*Myl2*) genes have decreased levels of transcripts and protein, altered myocardial histology, and reduced cardiac function (29), thereby supporting the functional importance of these sequences. Therefore, we aimed to investigate genetic elements that control *TTN* gene expression.

Here, we identified and tested potential *TTN* regulatory sequences through bioinformatics and experimental analyses using hiPSC-CMs and mouse models. We report a noncoding *TTN* element that promotes cardiac-specific expression and, when deleted, reduces *TTN* expression and impairs the contractility of CMs. We demonstrate cardiac TF binding sites including NK2 homeobox 5 (NKX2-5) and myocyte enhancer factor 2 (MEF2) motifs, which, when lost, abolished the *TTN* enhancer activity. We also characterized a rare human mutation that alters a predicted MEF2 site within this enhancer and downregulates *TTN* expression when introduced into hiPSC-CMs.

## Results

### TTN intron 1 is highly conserved and shows a euchromatic state.

**Figure 1:**
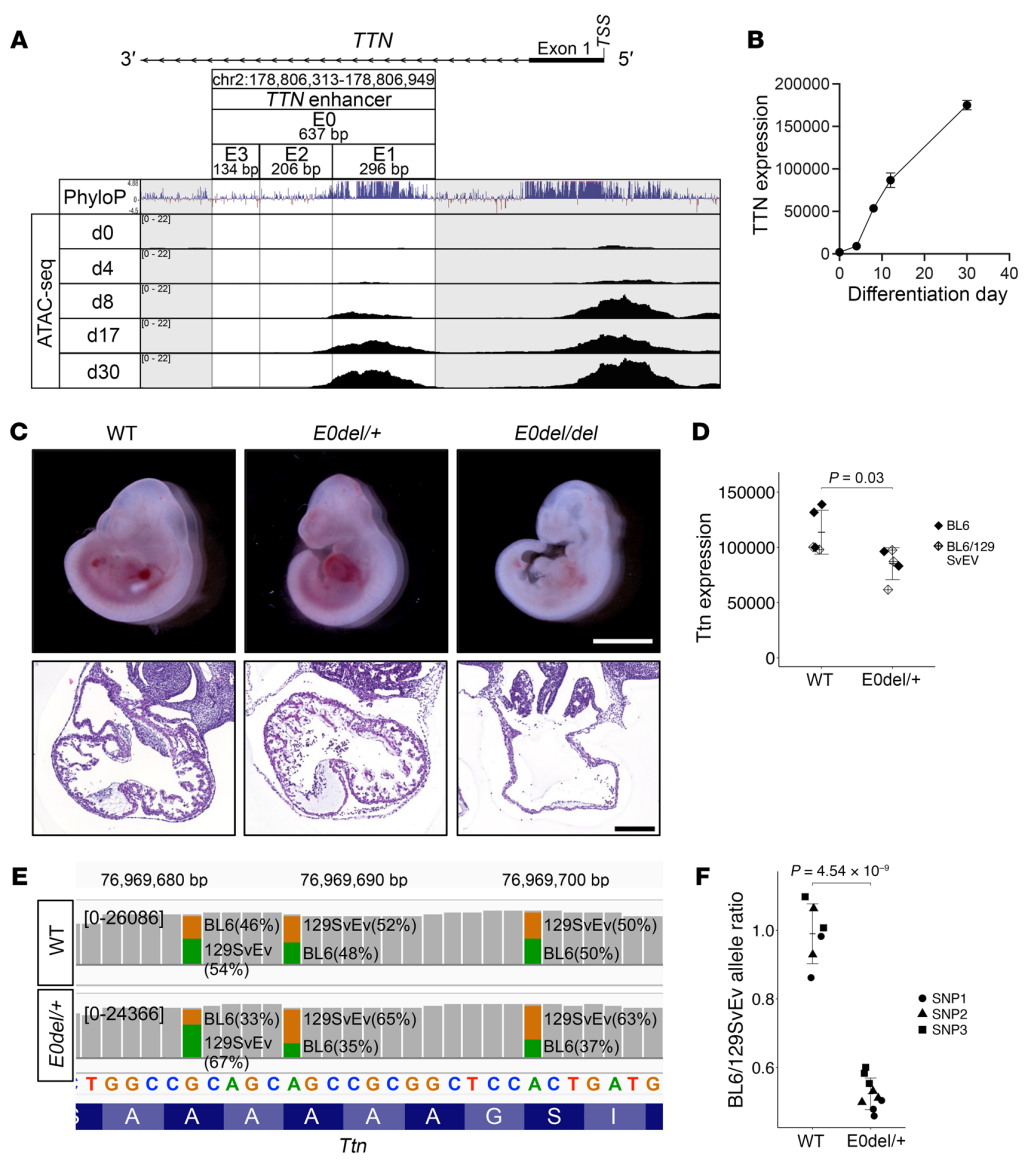
Identification of a TTN regulatory element. (**A**) Discovery of a highly conserved and euchromatic genomic region within intron 1 of TTN. A 637 bp DNA sequence within TTN intron 1 (E0) is highly conserved among vertebrates based on positive phyloP scores. ATAC-Seq library reads constructed from hiPSCs differentiating into CMs (from differentiation day 0 to day 30) demonstrated higher chromatin accessibility of the E0 region in more highly differentiated cells. Adapted from the UCSC human genome browser hg38 assembly (31). TSS, transcription start site. (**B**) TTN gene expression during hiPSC-CM differentiation. The *y* axis values indicate normalized counts derived from RNA-Seq analysis using the R package DESeq2 (66). Data were obtained from 2 independent differentiations. (**C**) Morphology of mouse embryos and hearts upon E0 deletion. E10.5 mouse embryos (top panel, scale bar: 2 mm) and H&E staining of hearts (bottom panel, scale bar: 200 μm) of mutant embryos and their WT littermates. (**D**) Bulk RNA-Seq analysis showing *Ttn* expression in LVs from 17-week-old *E0del/+* mice. *n* = 5 per genotype. The *P* value was calculated using an unpaired, 2-tailed *t* test with Welch’s correction. (**E**) Allele-specific expression of SNPs located within *Ttn*. *E0del/+* mice on the C57BL/6 background were crossed with WT mice on the 129SvEV background to enable assessment of relative expression of WT and E0del alleles. The *y* axis indicates the number of sequencing reads from MiSeq analysis. (**F**) Bulk RNA-Seq analysis of LVs from WT and *E0del/+* mice on the BL6/129SvEV hybrid genetic background demonstrated decreased expression of the allele carrying the E0 deletion. The *P* value was calculated using an unpaired, 2-tailed *t* test with Welch’s correction.

As a first step toward identifying *TTN* gene regulatory elements, we used CRISPR/Cas9 technologies to create homozygous deletion of an 18 kb fragment directly upstream of the *TTN* promoter sequence (hg38 chr2:178,807,594-178,826,116; Supplemental Figure 1A; supplemental material available online with this article; https://doi.org/10.1172/JCI183353DS1) in hiPSC-CMs. Analyses of *TTN* transcripts in WT and mutated hiPSC-CMs showed comparable levels of expression (*P* = 0.14; Supplemental Figure 1B). We then consulted the VISTA comparative genomics (30) and UCSC genome (31) browsers to identify other conserved sequences within 20,000 bp of the *TTN* transcriptional start site. A 637 bp sequence (hg38 chr2:178,806,313-178,806,949) within intron 1 of TTN (denoted E0) was highly conserved among 100 vertebrate genomes based on positive phyloP scores (32) and Multiz alignment tool (33) (Figure 1A and data not shown).

We determined whether the chromatin within the E0 region underwent dynamic remodeling by performing assays for transposase-accessible chromatin sequencing (ATAC-Seq) at days 0, 4, 8, 17, and 30 of CM differentiation from hiPSCs. A small ATAC-Seq peak, present at day 4 (cardiac mesoderm), subsequently increased (days 8–17) and was maintained after metabolic selection that enriched for CMs (day 30). The progressive heightening of chromatin accessibility correlated with increased *TTN* expression during CM differentiation (Figure 1, A and B) and H3K27 ChIP-Seq data from human and mouse hearts (34, 35) (Supplemental Figure 1C and Supplemental Figure 2A, respectively).

### Homozygous deletion of the putative Ttn enhancer is lethal in mice.

To elucidate the role of the putative *TTN* regulatory sequences, we used CRISPR/Cas9 to generate 3 independent mouse lines carrying the E0 deletion (denoted E0del, E0del2, and E0del3; Supplemental Figure 2B). As the genotypes of 141 offspring from heterozygous E0 crosses (22 litters) revealed no mice with homozygous E0 deletion (*P* = 1.61 × 10^–9^, χ^2^ test), we concluded that homozygous deletion of *Ttn* E0 sequences in mice was embryonically lethal (Table 1). *E0del/del* embryos were smaller than their WT and *E0del/+* embryos (Figure 1C) and were not viable after E10.5. Cardiac histology of E10.5 embryos demonstrated underdeveloped cardiac chambers with impaired trabeculation (Figure 1C). *E0del/+* mice were born at the expected Mendelian ratio, lived into adulthood, and were phenotypically normal with preserved cardiac function (Supplemental Table 1). Bulk RNA-Seq analyses of left ventricle (LV) tissues from WT and heterozygous mutant mice showed significantly decreased *Ttn* expression in *E0del/+* LVs (*P* = 0.03; Figure 1D). As *Ttntv/+* mice are phenotypically indistinguishable from WT mice, whereas *Ttntv/tv* mice are embryonically lethal (36), we examined the offspring of *Ttntv/+* and *E0del/+* matings. Compound *E0del/Ttntv* mice were embryonically lethal (Table 2). We interpreted these results to indicate that decreased expression of the WT *Ttn* allele, due to deletion of E0 sequences, disrupted a delicate balance of Ttn homeostasis in *Ttntv/+* mice that was sufficient for survival in unstressed conditions. Collectively, these findings support the idea that E0 sequences have putative enhancer properties that augment functional *Ttn* transcripts and protein to enable normal cardiac physiologic properties.

To determine whether E0 regulated *Ttn* expression in *cis* or in *trans*, we generated *E0del/+* mice on a hybrid genetic background by crossing C57BL/6 *E0del/+* mice and 129SvEv WT mice and assessed expression of strain-specific SNPs within *Ttn*. We performed next-generation sequencing analysis of *Ttn* transcripts from LV and skeletal muscle, where Ttn is highly expressed and compared the abundance of strain-specific *Ttn* SNPs. The *E0del* to WT allele ratio was significantly decreased from 0.99 ± 0.09 in C57BL/6/129SvEv WT mice to 0.52 ± 0.05 in C57BL/6/129SvEv *E0del/+* mice (*P* = 8.36 × 10^–6^; Figure 1, E and F, and Supplemental Table 2), indicating that E0 functions in *cis* to regulate *Ttn*.

### TTN regulatory elements drive gene expression in CMs.

**Figure 2:**
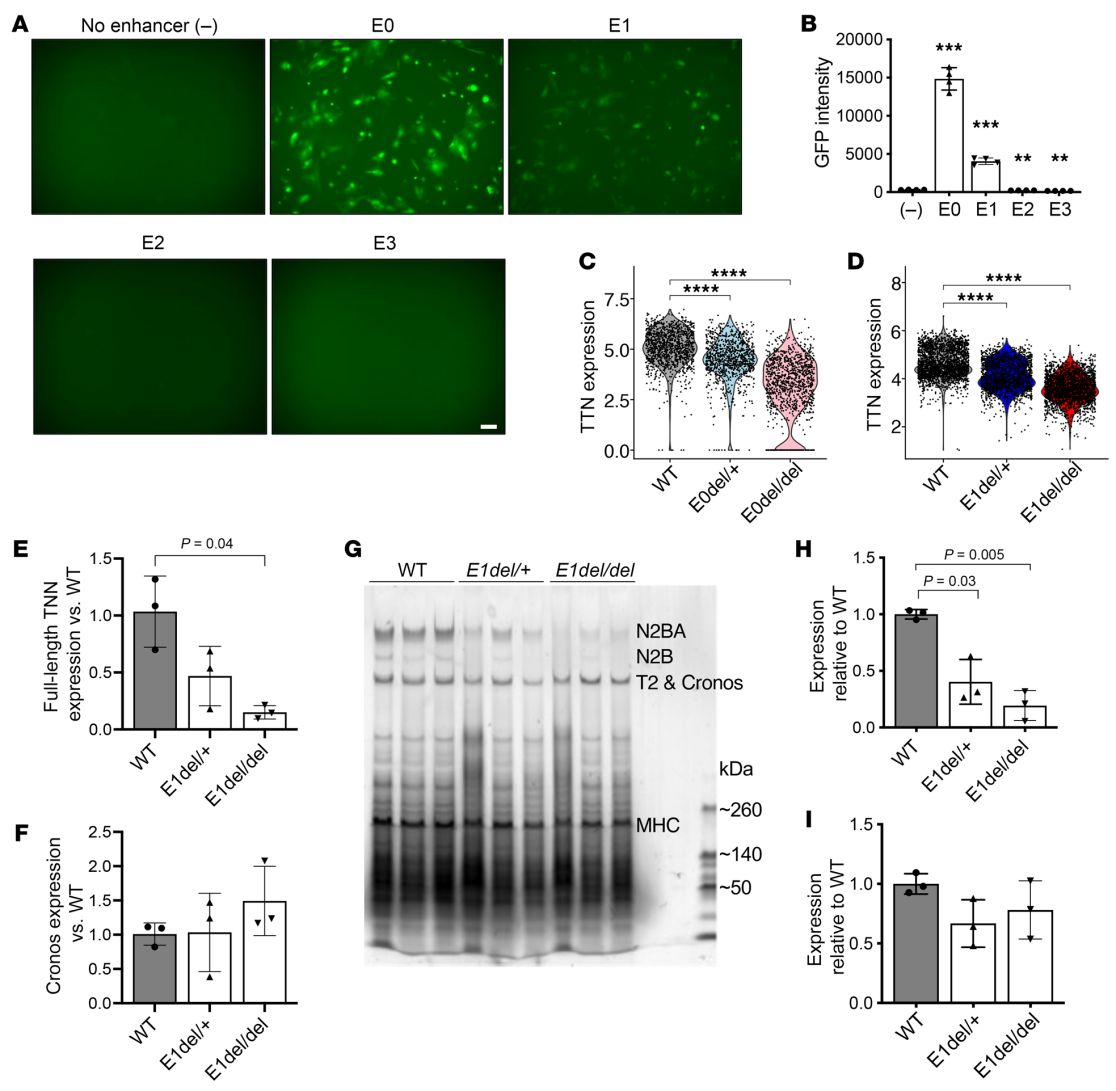
TTN regulatory elements are sufficient to drive gene expression in CMs and necessary for TTN expression. (**A**) GFP reporter gene assay in hiPSC-CMs. TTN regulatory elements E0 and E1 when inserted into a GFP expression vector with minimal promoter were able to induce GFP expression in hiPSC-CMs at differentiation day 30. In contrast, neither E2 nor E3 was able to activate GFP expression in hiPSC-CMs. Scale bar: 100 μm. (**B**) Quantification of GFP intensity by flow cytometric analysis. GFP intensity of hiPSC-CMs, which were transduced with lentivirus containing each TTN regulatory element, minimal promoter, and the GFP reporter gene, is shown. (–) indicates lentivirus containing minimal promoter and the GFP reporter gene. Data represent the mean ± SD. **P < 0.01 and ***P < 0.001, by unpaired, 2-tailed *t* test with Welch’s correction. TTN expression in hiPSC-CMs upon deletion of the TTN regulatory element E0 (**C**) and E1 (**D**). Multiplexed single nuclei RNA-Seq analysis was performed using hiPSC-CMs at differentiation day 30 from 2 and 4 independent differentiations, respectively. ****P < 0.0001, by unpaired, 2-tailed *t* test with Welch’s correction. Transcript level of full-length (**E**) and Cronos (**F**) isoforms of TTN. qPCR was performed in hiPSC-CMs at differentiation day 30. *n* = 3 per genotype, each set (WT, E1del/+, and E1del/del) from independent differentiations. TTN expression values normalized to WT are shown. *P* values were calculated using an unpaired, 2-tailed *t* test with Welch’s correction. (**G**) Total protein analysis of hiPSC-CMs using SDS-agarose gel stained with Coomassie blue. *n* = 3 per genotype, each set (WT, E1del/+, and E1del/del) from independent differentiations. Cell lysates were collected on differentiation day 30. Quantification of (**H**) N2BA and N2B and (**I**) T2 and Cronos TTN isoforms relative to WT. MHC was used for normalization. *P* values were calculated using an unpaired, 2-tailed *t* test with Welch’s correction.

We performed enhancer reporter assays to identify sequences within the putative enhancer that were sufficient to promote *TTN* expression in CMs. hiPSC-CMs were transduced with lentivirus carrying the GFP reporter constructs with human E0 sequences or one of the E0 segments: E1 (134 bp), E2 (206 bp), or E3 (296 bp) (Figure 1A). hiPSC-CMs transduced with the E0 fragment showed high levels of GFP fluorescence, unlike hiPSC-CMs transfected with either E2 or E3 fragments. E1 sequences also promoted robust GFP expression, albeit less than did E0, possibly reflecting differences in transduction efficiencies that were not assessed and/or the influence of sequences flanking the E1 fragment (Figure 2, A and B).

### Deletion of TTN regulatory elements abrogates TTN gene expression.

We assessed the effects of heterozygous (*E0del/+*) and homozygous (*E0del/del*) deletion of the E0 element in hiPSC-CMs using multiplexed single nuclei RNA-Seq analysis (37). To reduce potential variations in differentiation efficiency and cellular heterogeneity, only nuclei expressing mature CM marker genes (*TNNT2* and *MYH7*) were studied. We observed significantly reduced *TTN* transcript levels in mutant hiPSC-CMs lacking E0 compared with isogenic WT cells (Figure 2C and Supplemental Figure 3A).

We further studied the role of E1 sequences, the most conserved region within E0, by deleting E1 in TTN-GFP hiPSC-CMs. This cell line harbors GFP at the N-terminus of TTN and expresses normal transcript and protein levels of TTN (38). In comparison with isogenic WT cells, we found that *TTN* expression was significantly downregulated in both *E1del/+* and *E1del/del* hiPSC-CMs (*P* < 0.0001; Figure 2D and Supplemental Figure 3B). Parallel analyses of homozygous deletion of E1 in hiPSC-CMs derived from the WTC11 TTN-GFP hiPSC-CMs (Supplemental Figure 3C) confirmed these findings and excluded genetic background effects. Using quantitative PCR (qPCR), we also sought to determine whether the *TTN* enhancer E1 regulates the expression of full-length TTN or the shorter Cronos isoform. We found that E1 deletion reduced full-length *TTN* expression, but not Cronos transcript levels (Figure 2, E and F).

Consistent with transcriptional studies, analyses of total protein from hiPSC-CMs by SDS-agarose gel electrophoresis indicated that E1 deletion decreased TTN protein levels (Figure 2G). As full-length TTN protein isoforms (N2BA and N2B) were significantly reduced (*P* = 0.03 for heterozygous and *P* = 0.005 for homozygous mutant hiPSC-CMs; Figure 2H), while levels of the lower band (T2 and Cronos) were unchanged (Figure 2I), we concluded that E1 predominantly regulated expression of the full-length TTN.

### TTN enhancer deletion impairs sarcomere formation and function in CMs.

**Figure 3:**
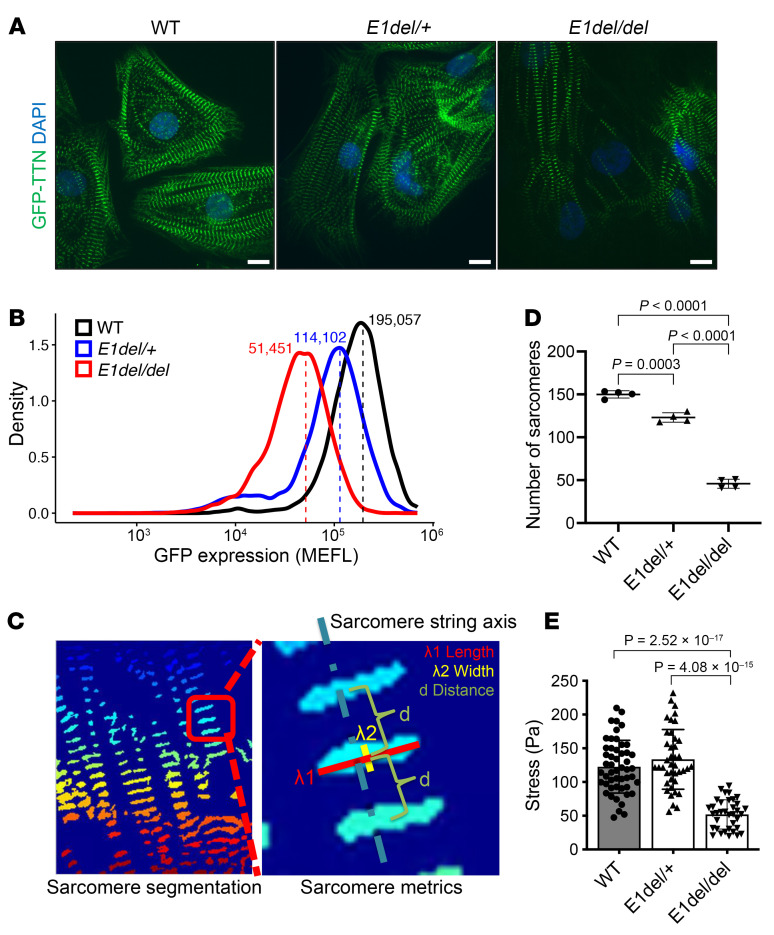
Deletion of the *TTN* regulatory element E1 leads to impaired sarcomere development and contractility in CMs. (**A**) TTN-GFP hiPSC-CMs at differentiation day 30 using fluorescence microscopy. Scale bars: 10 μm. (**B**) Quantification of sarcomeres in hiPSC-CMs with E1 deletion. Flow cytometric analysis of TNNT2^+^ TTN-GFP hiPSC-CMs at differentiation day 30 revealed a dosage-dependent decrease in TTN-GFP with E1 deletion. Data are from 3 independent differentiations. (**C**) Sarcomere analysis. Sarcomere segmentation of hiPSC-CMs was performed to analyze sarcomere organization and structural integrity. Key metrics include sarcomere length (λ1), sarcomere width (λ2), and the distance (d) between individual sarcomeres (right). (**D**) Number of sarcomere in hiPSC-CMs with and without E1 deletion. CMs were collected at differentiation day 30 to day 35 from 4 independent differentiation sets. Each dot represents mean values of data collected from six 6 wells of a 384-well plate. *P* values were calculated using an unpaired, 2-tailed *t* test with Welch’s correction. (**E**) Contractility of CMTs generated using hiPSC-CMs with E1 deletion. Data were obtained from 2 independent differentiations of hiPSC-CMs. Stress (Pa) represents contractile force divided by area. Error bars represent the SD. *P* values were calculated using an unpaired, 2-tailed *t* test with Welch’s correction.

We analyzed sarcomere organization of hiPSC-CMs and contractile function in TTN-GFP hiPSC-CMs (39). *E1del/del* compared with WT hiPSC-CMs exhibited fewer sarcomeres and weaker GFP expression, while *E1del/+* hiPSC-CMs appeared similar to WT cells (Figure 3A). Quantification of GFP expression by flow cytometry indicated that both *E1del/+* and *E1del/del* hiPSC-CMs had dosage-dependent reductions in TTN-GFP (Figure 3B and Supplemental Figure 3, D and E) and in the number of sarcomeres (Figure 3, C and D). Moreover, sarcomere structure was abnormal (Figure 3C and Supplemental Figure 4). *E1del/del* hiPSC-CM sarcomeres were shorter and thinner than isogenic WT cells (Supplemental Figure 4). These data implied that loss of E1 impaired both the quantity and architecture of sarcomeres.

We also investigated the functional effect of E1 deletion using cardiac microtissues composed of WT, *E1del/+*, or *E1del/del* hiPSC-CMs by assessing contractile forces. WT and *E1del/+* hiPSC-CMs had comparable contractility, while biallelic loss of E1 caused a significant reduction in contractile force compared with WT (58% decrease, *P* = 2.52 × 10^–17^; Figure 3E).

### Potential involvement of NKX2-5 and MEF2 in E1 activity.

**Figure 4:**
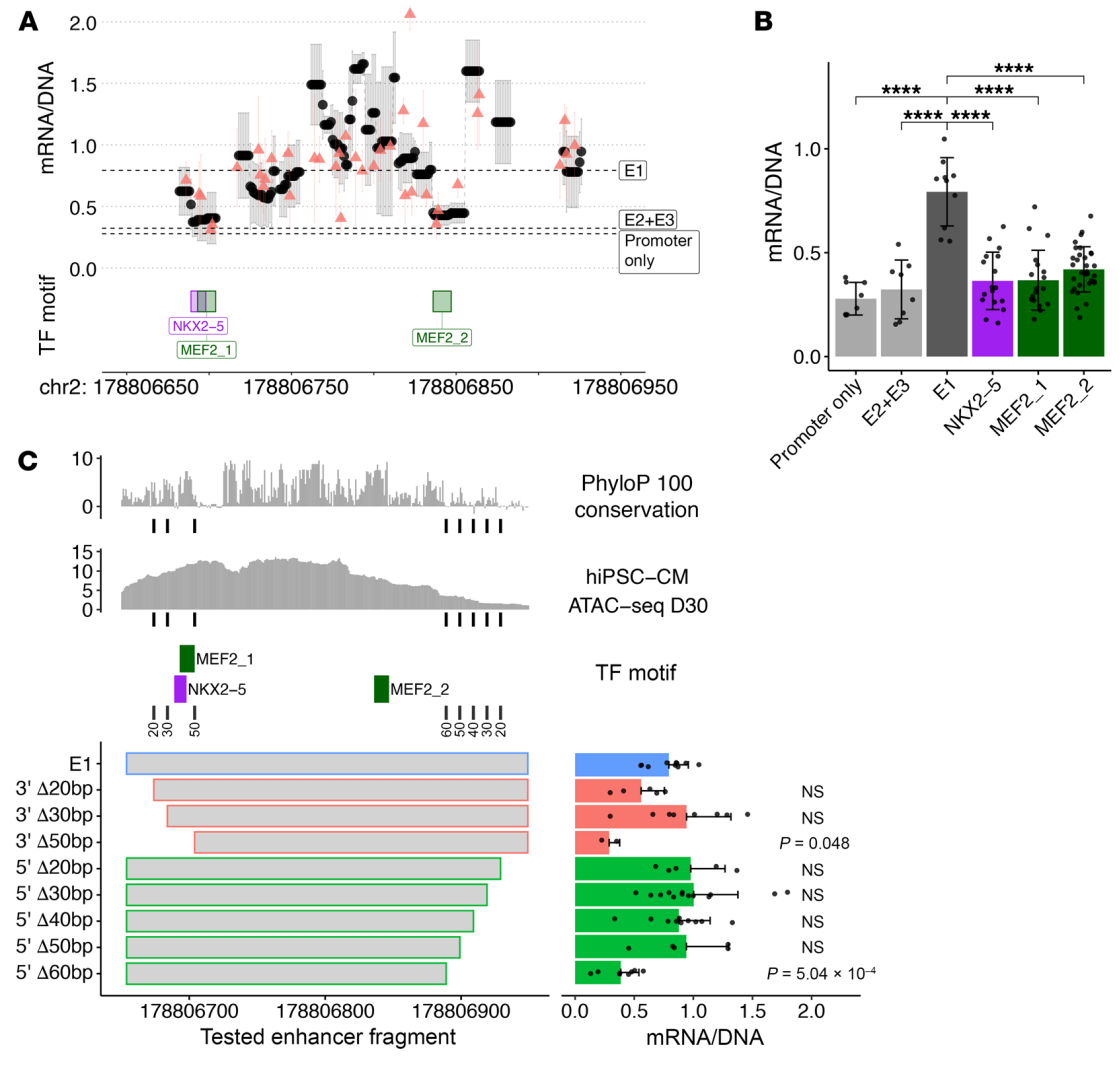
Sequence mutagenesis of E1 reveals an essential role of predicted NKX2-5– and MEF2-binding motifs in its transcriptional activity. (**A**) MPRA of mutated residues within E1 in hiPSC-CMs at differentiation day 30. The mRNA/DNA ratio indicates transcriptional activity when a nucleotide in that position is mutated. Black dots and red triangles represent deletion of a predicted TF binding site and point mutation to a specific nucleotide, respectively. GRCh38/hg38 genomic coordinates are noted at the bottom. (**B**) Predicted transcriptional binding motifs within E1 with a significant reduction in transcriptional activity upon mutagenesis. ****P < 0.0001, by unpaired, 2-tailed *t* test followed by Benjamini-Hochberg correction. Error bars represent the SD. (**C**) MPRA of E1 with serial deletions in its 5′ and 3′ ends. Conservation among 100 vertebrates and ATAC-Seq signal at hiPSC-CM differentiation day 30 for E1 are shown at the top. Predicted binding motifs for NKX2-5 and MEF2 described in **A** and **B** are noted in the middle. Error bars represent the SD. *P* values in **C** were calculated using an unpaired, 2-tailed *t* test followed by Benjamini-Hochberg correction.

We characterized E1 sequences that were critical for transcriptional activation using a modified massively parallel reporter assay (MPRA) protocol (40). hiPSC-CMs transfected with control plasmid (containing a minimal promoter), human E1, or E2+E3 sequences showed that E1 significantly increased the mRNA/DNA ratios compared with control and E2+E3 constructs (*P* < 0.0001; Figure 4, A and B). Using Hypergeometric Optimization of Motif EnRichment (HOMER) software (41), we defined predicted TF bindings motifs within E1 (Supplemental Table 3) and assessed transcriptional activities of constructs containing either a small deletion or a single nucleotide substitution in the most conserved nucleotides of each TF binding motif. The greatest decrement in E1 activity occurred in constructs that altered 2 regions within E1 (Figure 4, A and B), one with predicted binding motifs for NKX2-5 (*P* = 5.10 × 10^–6^) and MEF2 (*P* = 3.06 × 10^–6^; denoted as NKX2-5/MEF2_1), and one with a potential MEF2-binding motif (*P* = 2.51 × 10^–5^; denoted as MEF2_2). Previously published ChIP-Seq data in hiPSC-CMs and mouse hearts (42–44) demonstrated that these well-established cardiac TFs NKX2-5 and MEF2 bind E1 (Supplemental Figure 5).

We further generated 10–60 bp deletions of the terminal 3′ and 5′ sequences of E1 and tested the transcriptional activity using MPRA. Deletion of up to 30 bp from the 3′ end had no significant effect, whereas deletion of 50 bp that included the NKX2-5 and MEF2_1 sites significantly reduced the mRNA/DNA ratio (*P* = 0.048; Figure 4C). Deletion of 5′ sequences indicated that 50 bp was dispensable, whereas deletion of 60 bp significantly reduced E1 activity (*P* = 5.04 × 10^–4^; Figure 4C). As there were no predicted TF binding motifs within these deletion-sensitive 10 bp sequences, the mechanisms by which these sequences influence transcriptional regulation by E1 remain uncertain.

### Predicted MEF2- and NKX2-5–binding motifs are essential for in vivo cardiac-specific expression of the TTN enhancer E1.

**Figure 5:**
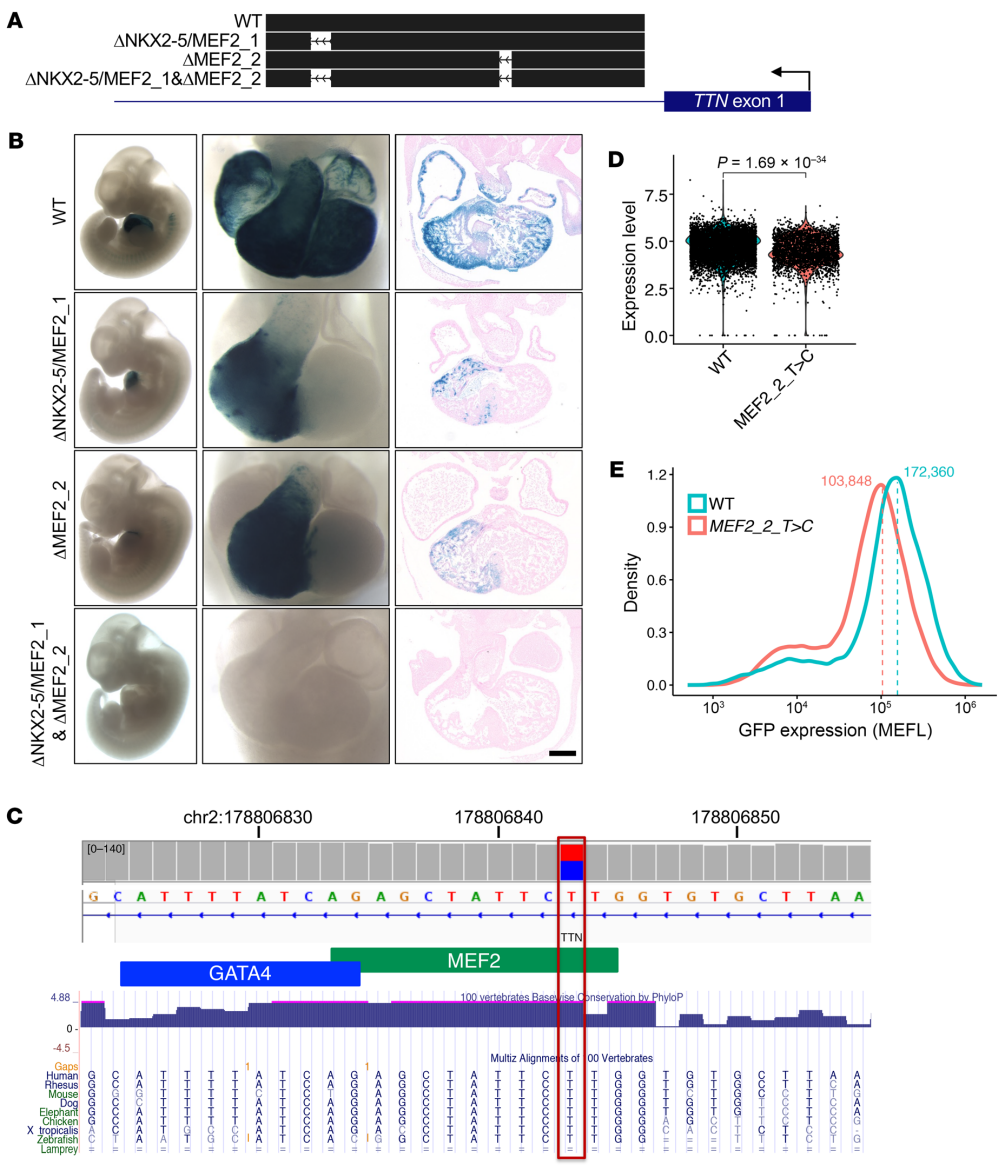
Predicted MEF2- and NKX2-5–binding motifs within E1 are critical for its transcriptional activity in vivo. (**A**) Schematic of the TTN enhancer constructs used for transgenic mouse reporter assay. (**B**) Transgenic mouse enhancer assay. Addition of human E1 sequences to the minimal *Shh* promoter was sufficient to induce transgene (β-gal) expression in a cardiac-specific manner, which was visualized as blue on a whole-mount staining of E11.5 mouse embryos. Mutation of the predicted MEF2- and NKX2-5–binding sites within E1 abrogated its expression. Expression pattern of the reporter gene was consistent among 5–8 transgenic mouse embryos within each construct group tested. Photos of representative embryos are shown in this figure. Scale bar: 200 μm. (**C**) A rare noncoding variant in E1 identified in a patient with DCM. Among 69 LV samples from patients with DCM studied by WGS, 1 DCM sample had a rare (MAF <2.00 × 10^–5^) variant within a highly conserved residue in a predicted MEF2-binding motif in E1 (chr2:178,806,843T>C; red box). Modified from the UCSC genome browser (31) and the Integrative Genomics Viewer (60). (**D**) TTN expression based on single nucleus RNA-Seq analysis. Each dot represents a single cell. The *P* value was calculated by Wilcoxon rank-sum test and adjusted for multiple testing. WT: 5 replicates from 3 differentiations; MEF2_2_T>C: 4 replicates from 2 differentiations. (**E**) Flow cytometric analysis of TTN-GFP hiPSC-CMs harboring a biallelic MEF2_2T>C mutation. Composite data are from 4 independent CM differentiations.

We validated in vivo enhancer activity of the human E1 sequence in a mouse model system. Mouse embryos were injected with a transgene containing E1 sequences (Supplemental Table 5) upstream of a minimal *Shh* promoter and *lacZ* reporter gene (45), and β-gal staining was assessed at E11.5. The E1 sequence strongly promoted β-gal activity in the hearts of transgenic mouse embryos (Figure 5, A and B), indicating a shared regulatory effect of E1 activities in humans and mice and consistent with the finding that E1 drove gene expression in hiPSC-CMs (Figure 2, A and B).

We also studied the requirement of the predicted NKX2-5/MEF2_1 and MEF2_2 motifs in E1 by generating reporter constructs carrying a deletion of the NKX2-5/MEF2_1 motif, the MEF2_2 motif, or both (Figure 5A and Supplemental Table 5). Mice transgenic for a reporter construct in which both the NKX2-5/MEF2_1 and MEF2_2 motifs were deleted showed no β-gal expression in the heart (Figure 5B). When only one of these motifs was deleted, the transgene activity was abolished in the LV but not in the right ventricle (RV) (Figure 5B). While the mechanisms by which these sites contribute to the observed chamber-specific effect remain to be determined, these results demonstrate that both sites were required for biventricular in vivo activity of the E1 enhancer. Overall, our findings indicate that the predicted NKX2-5– and MEF2-binding motifs were crucial for the activity of the E1 sequences in both hiPSC-CMs and mouse hearts.

### Genome sequencing analysis identifies an E1 mutation in a patient with DCM.

Prompted by experimental evidence that intron 1 sequences regulate *TTN* expression, we considered whether genetic variants within this region might contribute to DCM in humans. From whole-genome sequencing (WGS) analysis of 69 cardiac tissues from patients with unexplained DCM, we identified 1 rare genetic variant (minor allele frequency [MAF] <2.00 × 10^–5^ in gnomAD) within E1. Variant chr2:178,806,843T>C, located within the predicted MEF2_2 motif, was present in 1 of 2 patients with DCM of African ancestry. The variant frequency in these DCM patients compared with racially matched gnomAD reference individuals was unlikely to arise by chance (*P* = 3.86 × 10^–4^); however, the small size of the available cohort may limit the statistical significance. Notably, this variant resides within highly conserved E1 residues and alters a predicted MEF2-binding motif residue that is highly conserved among 100 vertebrates (Figure 5C).

We introduced the variant chr2:178,806,843T>C into TTN-GFP hiPSCs via homology-directed repair (HDR) and differentiated them into CMs. We performed single nucleus RNA-Seq analysis and flow cytometry to quantify TTN expression. hiPSC-CMs with biallelic MEF2 binding site variants (MEF2_2_T>C) had decreased TTN expression in comparison with isogenic WT cells at both the transcript and protein levels (Figure 5, D and E, and Supplemental Figure 6, A, and C). We additionally measured the force generated by MEF2_2_T>C cardiac microtissues and observed decreased contractility (Supplemental Figure 6C).

## Discussion

We identified highly conserved sequences (denoted E1) that regulate the transcription of *TTN*, an essential CM gene that harbors pathogenic variants in patients with DCM and heart failure. Deletion analyses demonstrated that the E1 sequences within this element are necessary for *TTN* expression and normal CM function. Ablation of this noncoding element impaired sarcomere development and markedly decreased CM contractility, thereby providing further support that TTN haploinsufficiency contributes to cardiac dysfunction that occurs in patients with DCM with truncating TTN variants. We also show that E1 was sufficient to drive *TTN* expression in hiPSC-CMs and CM expression in the mouse. Together, our findings support the conclusion that the highly conserved region in intron 1 of *TTN* is a critical regulatory element of *TTN*.

The sequences contained within introns of human, *Drosophila*, and plant genes are conserved and enriched for active transcriptional regulatory signals, particularly among large multidomain genes (46). Intronic enhancers can influence epigenetic regulation to increase the expression of the gene they are located in or a neighboring gene up to 100-fold (46, 47). The location of E1 within intron 1 and high sequence conservation, combined with our experimental evidence for its epigenetic activation during CM differentiation, cardiac-specific transcriptional effects in mice, and *TTN* expression in hiPSC-CMs, provides strong evidence that E1 is a *TTN* enhancer.

Deletion of 1 copy of the *Ttn* enhancer E0 in mice led to a decrease in Ttn expression by 25% (Figure 1D) but did not affect cardiac function assessed by echocardiography (Supplemental Table 1). By contrast, homozygous deletion of E0 caused embryonic lethality (Table 1). Similarly, *Ttntv/+* mice are phenotypically normal, but *Ttntv/Ttntv* die in utero. Furthermore, our analysis of hiPSC-CMs suggests that *E0del/del*, but not *E0del/+*, hiPSC-CMs had significantly impaired contractility (Figure 3E). *TTN* expression was decreased by 35% in *E0del/+* hiPSC-CMs (Supplemental Figure 3A) and by 75% in *E0del/del* hiPSC-CMs (Supplemental Figure 3B). TTN is an essential sarcomere protein, but it is currently unknown how much is necessary for mammalian development and life-long function. Given the results of our studies of TTN expression in hiPSC-CMs and mice, we suggest that *Ttn*-mutant mice require biallelic levels that are 25% or higher than those found in WT mice.

Our MPRA findings (Figure 4), published ChIP-Seq data from hiPSC-CMs and mouse heart (Supplemental Figure 5), and transgenic reporter assays in mice (Figure 5) support the conclusion that *NKX2-5* and *MEF2* regulate E1 activities to promote *TTN* transcription. The pivotal roles of *NKX2-5* and *MEF2* in cardiac development are well established. Deletion of *Nkx2-5* in mice results in lethality at E9.5 with abnormal cardiac morphogenesis (48). *Mef2* is essential for the expression of cardiac-specific genes including α-myosin heavy chain (49, 50). In addition to these, other binding motifs within E0 sequences (Supplemental Figure 5 and Supplemental Table 3) indicate the potential activities by other key cardiac TFs, including GATA binding protein 4 (*GATA4*), T-box transcription factor 5 (*TBX5*), serum response factor (*SRF*), and TEA domain transcription factor 1 (*TEAD1*) (42–44). As cardiac TFs physically interact with each other and work in synergy to modulate the transcriptional activity of cardiac-specific genes, we suggest that these may cooperate to influence E1 activities (51–54).

It is unclear why deletion of a single predicted MEF2-binding motif is sufficient to selectively abolish transgene expression specifically in the LV (Figure 5B). Previous studies have shown that homozygous null mutations in *Mef2c* leads to absence of the RV (49). Isl1 and GATA factors, which play a crucial role in the development of the second heart field, a population of cardiac progenitor cells contributing to the outflow tract, the RV, and parts of the atria (55, 56) are known regulators of *Mef2c* expression (57). Earlier studies identified noncoding elements activating *Mef2c* expression in the RV, outflow tract, and ventricular septum (57, 58). Hence, higher *Mef2c* expression in the RV could plausibly allow persistent transgene expression despite the lack of a single binding motif. Alternatively, we speculate that, unlike in the RV, MEF2 regulation of gene expression in the LV involves distinct TFs.

Notably, deletion of 60 bp at the 5′ end of E1, but not 50 bp, led to a significant reduction in transcriptional activity of E1. These 10 bp may contain uncharacterized TF binding motifs or perhaps provide an optimal physical conformation for TFs that bind nearby motifs or that interact with transcriptional coactivators to activate gene expression. Expanded analyses of sequences that are not contained in the E1 fragment may help to explain this observation.

We propose that WGS will provide further insights into regulatory elements involved in the expression of genes that cause DCM by haploinsufficiency. From a small cohort of 69 samples from patients with unexplained DCM, we identified 1 rare variant within the highly conserved predicted MEF2-binding motif in the E1 fragment (Figure 5C). While preliminary, this finding supports the importance of E1 sequences in regulating *TTN* and thereby contributing to DCM. We predict that further genome sequencing of unexplained DCM will help to decipher the roles of NKX2-5 and MEF2 in the transcriptional regulation of *TTN* and, more broadly, the potential for noncoding regulatory variants to cause or contribute to DCM that remains unresolved in many patients.

*TTNtv* account for approximately 20% of human DCM cases (9, 59) and increase susceptibility to DCM from environmental stress such as pregnancy (14), alcohol (15), and cancer therapy (16). Increasing the amount of the TTN protein that is derived from the normal allele would likely benefit these patients. Identifying essential regulatory elements that regulate *TTN* expression is critical to advancing this goal. We show that E1 is an enhancer that is necessary and sufficient for *TTN* expression in cultured CMs and in in vivo myocardium and enables sarcomere development and physiological functions. Further characterization of endogenous signals that activate this enhancer and small molecules or genetic activation of E1 may provide new opportunities to increase *TTN* expression levels and overcome the deleterious effects of TTNtv that cause DCM and cardiac arrhythmias.

## Methods

### Sex as a biological variable.

For animal studies involving mice with E0 deletion, both male and female animals were used, and similar findings are reported for both sexes.

### Generation of mice with E0 deletion.

We designed the guide RNA1 (gRNA1) and gRNA2 targeting the 5′ and 3′ ends of the E0 sequences (Supplemental Table 4) and prepared the ribonucleoprotein (RNP) complex (gRNAs and Cas9 nuclease) to be injected into C57BL/6J mouse zygotes (The Jackson Laboratory). After injection, we screened founders for the enhancer deletion and backcrossed them with C57BL/6 mice to obtain mice on a pure background. We performed PCR with sequencing confirmation using MiSeq analysis and Integrative Genomics Viewer (60) review to establish F0 (mosaic), PCR followed by MiSeq to determine F1, and then qPCR by Transnetyx (Cordova, Tennessee, USA) to genotype subsequent offspring. The gRNA sequences are listed in Supplemental Table 4.

### Assessment of cardiac function in the mouse model system.

Cardiac function was assessed using a digital ultrasound system (Vevo F2 Imaging System and UHF57x transducer, FujiFilm VisualSonics) by an experienced observer blinded to the mouse genotype. Mice were anesthetized under an isoflurane vaporizer (VetEquip) and were placed on the heating table with ECG leads. Sedation was lightened after a mouse was positioned properly for imaging, and all measurements were performed with a heart rate between 500 and 550 beats per minute. 2D and M-mode images of the LV (parasternal long axis and short axis) were obtained. Measurements were averaged from 3 consecutive heartbeats of M-mode tracings that included left ventricular end-diastolic (LVEDD) and end-systolic (LVESD) chamber dimensions, interventricular septal (IVS) thickness and left ventricular posterior wall (LVPW) thickness. Left ventricular fractional shortening (FS) was calculated as follows: (LVEDD – LVESD)/LVEDD *×* 100%.

### hiPSC culturing and CM differentiation.

hiPSCs used in this study were derived from the male parent hiPSC line PGP1 (61). We also engineered a commercially available TTN-GFP hiPSC line on the WTC11 background (Allen Institute) to study the function of the TTN enhancer E1 on a different genetic background. The cells were cultured on Matrigel-coated plates (BD Sciences) in mTeSR1 media (STEMCELL Technologies). The hiPSC-CMs were generated from hiPSCs using a small-molecule–mediated differentiation method, which modulates the Wnt signaling pathway, and CMs were metabolically selected by glucose deprivation as previously described (39).

### ATAC-Seq analysis.

ATAC-Seq was performed as previously described (62). Briefly, approximately 50,000 cells were harvested and lysed to isolate nuclei, which were treated with Tn5 transpose (Nextera DNA Sample Prep Kit, Illumina). Fragmented DNA was amplified using bar-coded PCR primers, and pooled libraries were sequenced to a depth of 10 million reads per sample (Illumina Next-Seq). Sequencing reads were aligned to the hg38 reference genome using BWA-MEM, and peaks were called using HOMER (version 4.9) (41).

### Enhancer reporter assay in hiPSC-CMs.

DNA fragments containing sequences of the TTN regulatory elements E0, E1, E2, and E3 were synthesized by Twist Bioscience and subcloned into pLS-mP (Addgene, no. 81225) using the NEBuilder HiFi DNA Assembly Kit (New England Biolabs, E2621S). These TTN enhancer plasmids, together with the VSV-G envelope–expressing plasmid pMD2.G (Addgene, no. 12259) and the lentiviral packaging plasmid psPAX2 (Addgene, no. 12260), were cotransfected into HEK293T cells to produce lentivirus. The supernatant of lentivirus was concentrated by a Lenti-X concentrator (Takara, 631232) and quantified by qPCR using the Lentivirus Titration Kit (Applied Biological Materials, LV900). To test TTN enhancer activity, hiPSC-CMs at differentiation day 30 were transduced with each TTN enhancer lentivirus (MOI = 6). Six days after transfection, the infected hiPSC-CMs were analyzed by flow cytometry, and the mean GFP fluorescence intensity was calculated to assess enhancer activity. Four transfections were done per construct.

### CRISPR/Cas9 gene editing.

*18kb del/+, E0del/+*, and *E0del/del* hiPSCs were generated using nonhomologous end-joining by cotransfection of 2 μg plasmid expressing Cas9 (PX459v2, Addgene, no. 62988) and 2 μg plasmid expressing gRNA using a stem cell nucleofector kit (Amaxa). Edited clones were selected using Puromycin and subcloned by placing individual monoclonal hiPSC colonies into separate, individual wells of a 96-well plate. *E1del/+* and *E1del/del* hiPSCs were generated by transfection of RNP complexes consisting of CRISPR RNA (crRNA) targeting each end of the E1 sequences, trans-activating CRISPR RNA (tracrRNA), and Cas9 protein. RNP was delivered into the cells using Amaxa 4D nucleofector with program CB150 (Lonza). The cells were plated at a low density and cultured until monoclonal hiPSC colonies were visible. Clones were then isolated and moved to a 96-well plate. DNA was isolated from the clones, amplified using PCR, and sent for Sanger sequencing to assess for zygosity. The PCR amplicons were submitted for next-generation sequencing to confirm their genotypes. hiPSCs harboring a homozygous T-to-C mutation in the MEF2_2 motif within E1 were generated by transfection of the RNP complex and 1 μL of 100 μM single-stranded HDR template carrying the T-to-C mutation. The gRNA sequences used for CRISPR/Cas9 gene editing are listed in Supplemental Table 4.

### Multiplexed single nuclei RNA-Seq analysis.

hiPSC-CMs were replated with 10× TrypLE after glucose starvation to remove potential cell debris. At differentiation day 30, hiPSC-CMs were dissociated with 10X TrypLE. Cells were centrifuged and washed with PBS. CM pellets (1 × 10^6^ to 2 × 10^6^ per replicate) were collected and frozen at –80°C. Multiplexed single nuclei RNA-Seq was conducted as previously described (37). In short, cells were lysed, and the isolated nuclei were incubated with the commercially available lipid-oligo barcodes (3′ CellPlex Kit from 10X Genomics). Nuclei were washed, counted, and pooled at equal numbers. Intact single nuclei were sorted with FACS. The standard 10X Genomics sequencing was performed according to the manufacturer’s instructions. CellBender (63) was used to remove nonspecific RNA expression. Aligned data were deconvoluted using the lipid-oligo barcode sequences and further analyzed using Seurat. The gene expression matrix was generated by *Read10X* of the Seurat package. Nuclei passing the following quality control metrics were kept for the downstream analysis: 25,000 > nCountRNA > 500, 6,000 > nFeatureRNA > 300, 5 > percent_mito, 0.5 > solo_score, and 0.3 > scrublet_score (64, 65). Reads were normalized using the standard Seurat commands. Differential gene analysis was conducted using 2 different methods. Initial differential gene analysis was done using FindMarkers with the default Wilcoxon rank-sum test. Cutoffs for log fold change changes and minimum cell percentage were set to 0.25. All *P* values were adjusted for multiple testing based on the Benjamini-Hochberg correction on all genes in the dataset. Pseudobulk differential gene analysis was conducted using DESeq2 (66) by aggregating gene expression at the sample level.

### qPCR.

Total RNA was manually isolated from frozen cell pellets using the traditional TRIzol method (Thermo Fisher Scientific). cDNA was synthesized using the SuperScript III First-Strand Synthesis System (Invitrogen, Thermo Fisher Scientific) following the manufacturer’s protocol and using 100 ng total RNA per reaction. qPCR was performed on a 7500 Fast Real-Time PCR System (Applied Biosystems) using 10 μL Fast SYBR Green Master Mix (Invitrogen, Thermo Fisher Scientific), 1 μL of each primer in the pair (5 μM), 2 μL cDNA, and 6 μL double-distilled H_2_O (ddH_2_O). Thermocycle programs consisted of 40 cycles each, with 3 seconds at 95°C and 30 seconds of annealing/extension at 60°C. Relative expression levels were determined using the ΔΔCt method with *HPRT* as the internal housekeeping gene. PCR primers were designed on the basis of a previously published protocol (67), which included FullTTN_F (5′-TTTTGCACAACTGTCGCCTG-3′); Cronos TTN_F (5′-ACGCAAAGCTGTTTCTTCCC-3′); CronosFullTTN_R (5′-CTTCGTAGGAGAGC TCGCAG-3′); HPRT_F (5′-TGACACTGGCAAAACAATGCA-3′); and HPRT_R (5′-GGTCCTTTTCACCAGCAAGCT-3′).

### hiPSC-CM imaging via confocal microscopy.

hiPSC-CMs were replated on a 12-well, glass-bottomed culture plate (MatTek, P12G-1.0-10-F) coated with Matrigel (Corning, 354277) at differentiation day 20. Cells were then fixed at differentiation day 30 with 4% paraformaldehyde (PFA) in PBS for 10 minutes and washed 3 times with 2 mL PBS at room temperature. PBS (1 mL) containing 2 drops of NucBlue Fixed Cell Stain ReadyProbes reagent (DAPI) (Thermo Fisher Scientific, R37606) was added to each well for 10 minutes and then washed off 3 times with 2 mL PBS at room temperature.

Imaging was performed on a Nikon Ti inverted W1 Yokogawa Spinning disk microscope with a 50 μm pinhole disk and an Andor Zyla 4.2 Plus sCMOS monochrome camera. Nikon Elements Acquisition Software AR 5.02 was used to acquire single Z-plane 16-bit ND2 images via a Plan Apo Lambda 100× oil immersion objective with a numerical aperture of 1.45. Laser lines 405 and 488 were used to excite DAPI-stained nuclei and TTN-GFP, respectively. For every region of interest, multiple Z-plane images were taken at 0.5 μm intervals spanning the full height of the cells in order to capture the full 3D structure. Fiji (version 2.15.1) (68) was used to compile all Z-plane images for each region of interest into a maximum intensity Z projection.

### Flow cytometric analysis.

hiPSC-CMs were detached from a 6-well plate by incubation with 500 μL TrypLE Select Enzyme (10x), no phenol red (Thermo Fisher Scientific, A1217701) at 37°C for 6 minutes, followed by resuspension in 1 mL RPMI (Thermo Fisher Scientific, 61870127) supplemented with B-27 (Thermo Fisher Scientific, 17504044). Resuspended cells were centrifuged at 300*g* for 5 minutes. The supernatant was suctioned away from the cell pellet, and cells were resuspended in 1 mL PBS with 0.5% BSA by pipetting. Cells were passed through a 70 μM cell strainer to remove large clumps.

Cells were then fixed with 4% PFA in PBS for 30 minutes at room temperature and washed 3 times with 0.5 mL PBS. Between each wash, cells were centrifuged at 300*g* for 5 minutes. Cells were then permeabilized with 0.5 mL 0.2% Triton X-100 in PBS for 20 minutes at room temperature on a rocker. After centrifuging at 300*g* for 5 minutes and aspirating the supernatant, cells were blocked in 0.5 mL PBS containing 5% BSA and 0.05% Triton X-100 for 2 hours at room temperature followed by centrifugation at 300*g* for 5 minutes, and the supernatant was then removed. Cells were incubated overnight at 4°C with 0.5 mL PBS containing 5% BSA, 0.05% Triton X-100, and rabbit cardiac troponin T antibody (Abcam, AB45932, 1:200). The cells were then washed and centrifuged 3 times using 0.5 mL PBS containing 5% BSA and 0.05% Triton X-100. Cells were stained for 4 hours at room temperature with 0.5 mL PBS containing 5% BSA, 0.05% Triton X-100, and donkey anti–rabbit IgG Alexa Fluor 568 (Abcam, AB175470, 1:500). The cells were again washed and centrifuged 3 times using 0.5 mL PBS containing 5% BSA and 0.05% Triton X-100 prior to flow cytometry (BD FACSAria). A sample of rainbow calibration particles (Spherotech, RCP-30-5A) was also collected on the same channel for each day and time point.

Flow cytometric data were processed using FlowJo software. Briefly, live cells were gated according to size and morphology from a forward scatter area (FSC-A) versus side scatter area (SSC-A) scatter plot, and singlet cells were further isolated from the live-cell population by grouping cells along the diagonal of an SSC-A versus SSC-W, and then a FSC-A versus FSC width (FSC-W) scatter plot. Finally, TNNT2^+^ cells that displayed high DAPI signal (Pacific Blue channel) in a scatter plot of Pacific Blue-A versus FSC-A were selected and analyzed. GFP AU were converted to standard mean equivalent fluorochrome (MEFL) units to reduce day-to-day variability in the fluorescence data collected.

### Sarcomere analysis.

Imaging was performed on a Yokogawa CV7000 microscope in scanning confocal mode using a dual Nipkow disk. Plates (384-well) (Perkin Elmer, 6007558) were mounted on a motorized stage, and images were acquired in a row-wise zigzag fashion at room temperature for fixed cells. The system’s CellVoyager software and 405, 488, 561, and 640 nm solid laser lines were used to acquire single Z-plane 16-bit TIFF images through a dry 40× objective lens using a cooled sCMOS camera with 2,560 × 2,160 pixels and a pixel size of 6.5 μm without pixel binning. Nine images in a 3 × 3 orientation were acquired from the center of each well. Image segmentation and feature extraction were performed with the C++ in-house software PhenoLink toolsuite (Github) and the SarcoKSI image processing plugin. Image segmentation was performed on illumination-corrected raw images based on GFP channel intensity thresholds. The SarcoKSI plugin involves the segmentation of filaments from dedicated image channels (e.g., GFP-TTN). For each filament, λ1 and λ2 (representing the primary and secondary axes of the filament, respectively), d (filament-to-filament distance, from center to center), filament area, and the angle of the filament string relative to the vertical axis were measured (Supplemental Figure 4). For each group, we calculated statistical significance using an unpaired *t* test with Welch’s correction.

### Protein gel analysis.

hiPSC-CMs at differentiation day 30 were washed, thoroughly aspirated, and lysed directly in the well with 2 M thiourea, 3% SDS, 50 mM Tris (pH 6.8), and 75 mM freshly added dithiothreitol and stored at –80°C. Samples were then recombined with 1 volume of 1:1 glycerol with protease inhibitors and triturated with a pipette until samples were able to be pipetted for electrophoresis.

The prepared total protein was used for SDS-agarose electrophoresis as previously described (69). The running buffer was 50 mM Tris and 0.384 M glycine (pH 8.3). Gels were cast from running buffer containing 1% Sea-Kem Gold agarose (Lonza) plus 30% glycerol. The gel was retained within the plates by a folded segment of nylon mesh placed between the plates. Fresh 2-mercaptoethanol was added to the upper buffer chamber at a dilution of 1:1,000 just before sample loading. The gel apparatus was nearly completely filled with prechilled running buffer and placed in a slurry of ice during the run, which was conducted at a constant current of approximately 16 mA per gel. For total protein staining of SDS-agarose electrophoresis gels, gels were stained with GelCode blue (Thermo Fisher Scientific) according to the manufacturer’s instructions and imaged on a LiCor Odyssey CLX infrared imager at 700 nm. Band quantification was done using Image Lab software (Bio-Rad) and normalized to MHC.

### Cardiac microtissue generation and contractility analysis.

Cardiac microtissues (CMTs) were created by seeding cells into 6-well plates containing 2 micro-pillars (spring constant = 2.68 μN/μm) casted from a 3D printed mold (Protolabs) as previously described (70). The devices were plasma treated for 60 seconds, followed by treatment with 0.01% poly-l-lysine (ScienCell) for 1.5 hours and then 0.1% glutaraldehyde (Electron Microscopy Sciences [EMS]) for 15 minutes, washed 3 times with deionized (DI) water, and finally soaked in DI water at 4°C until use. Immediately prior to seeding, the devices were soaked in 70% ethanol for 15 minutes, dried, and sterilized under ultraviolet light for 15 minutes. After sterilization, 2% Pluronic F-127 (MilliporeSigma) was added to each well below the pillar caps and incubated for 30 minutes at room temperature to prevent CMTs from attaching to the bottom surface of the polydimethylsiloxane devices.

A total of 60,000 cells were seeded per tissue, which consisted of 90% hiPSC-CMs and 10% cardiac fibroblasts (Lonza, CC2904). Cells were delivered in 4 mg/mL human fibrinogen (MilliporeSigma), 10% Matrigel (Corning), 0.4 units of thrombin (MilliporeSigma) per milligram of fibrinogen, 5 μM Y-27632 (Tocris), and 0.033 mg/mL aprotinin (MilliporeSigma). After gel polymerization for 10 minutes, CMTs were cultured in tissue maintenance growth media (high-glucose DMEM, Thermo Fisher Scientific) supplemented with 10% FBS (MilliporeSigma), 1% penicillin-streptomycin (Thermo Fisher Scientific), 1% Nonessential Amino Acids (Thermo Fisher Scientific), 1% GlutaMAX (Thermo Fisher Scientific), and 0.033 mg/mL aprotinin), with the addition of 5 μM Y-27632 from day 0 to day 2. Tissue maintenance growth media were replaced every other day.

On day 7, time-lapse videos of the tissue contraction were acquired at 30 frames per second using a 4× objective on a Nikon Eclipse Ti (Nikon Instruments) with an Evolve EMCCD Camera (Photometrics), equipped with a temperature and CO_2_ equilibrated environmental chamber. CMTs were electrically stimulated at 1 Hz using a C-Pace EP stimulator (IonOptix). A custom MATLAB script (71) was used to measure the deflection of the pillars through edge tracking in 2 stages, first with pixel-scale cross-correlation followed by refinement at a subpixel resolution. Maximum contractile force and stress were then calculated on the basis of the tracked deflection of the pillars from the resting position and the measured pillar spring constant of 2.68 μN/μm (70, 72).

### MPRA.

A total of 65 potential TF binding sites within E1 were identified using HOMER (41). Constructs harboring deletion of the predicted TF binding sites (55 constructs) or point mutations of the most conserved residue within each TF binding motif (65 constructs) were designed and synthesized by Twist Bioscience. Constructs harboring serial deletions at the 5′ or 3′ end of the E1 fragment were also generated by Twist Bioscience. To minimize occurrence of the restriction enzyme site in the gene fragments, *SalI* was substituted for *XbaI* when cloning the inserts. To accommodate this change, we mutated the *SalI* site downstream of the polyA signal in pMPRA1 (Addgene no. 49349) using *MfeI* and *BbsI* sites in proximity. Pooled fragments were subcloned following the published MPRA protocol (40). The pooled library was digested with *Sfi1* and then cloned into the modified pMRPA1 backbone. Ligated plasmids were transformed into 5-α competent *E*. *coli* (New England Biolabs) and harvested using a Qiagen Maxiprep kit. Minimal promoter and luciferase sequences isolated from pMPRAdonor2 (Addgene no. 49353) were inserted using *Kpn1* and *Sal1* sites. The final plasmid library was concentrated with ethanol and verified using Sanger sequencing.

Each construct was introduced into 3 independent libraries and transfected into 4 wells of hiPSC-CMs (differentiation day 30) with Lipofectamine 3000 (Thermo Fisher Scientific). Forty-eight hours after transfection, polyA RNA was harvested using the Dynabeads mRNA DIRECT Kit (Thermo Fisher Scientific). To ensure that there was no carryover of plasmid DNA, harvested mRNA was treated with DNase 1, and cDNA was synthesized using the SuperScript III First Strand Synthesis System (Thermo Fisher Scientific) with oligo dT according to the manufacturer’s instruction. The MPRA library was amplified from cDNAs and plasmids using TagSeq primers. Sequencing reads were first matched with plasmid sequences. Barcode reads were matched, counted, and normalized to the total number of barcode reads. Analyses excluded constructs with low barcode reads (<100 normalized counts) and technical replicates (*n* = 4) in which the WT signal was 1 or higher SD of the mean. In the final analysis, data from 41 TF deletion constructs, 53 point mutation constructs, and 8 end-deletion constructs were included.

### Transgenic reporter assay in mouse models.

Transgenic enhancer reporter assays were performed per established protocols (45, 73). Briefly, a minimal *Shh* promoter and a reporter gene were integrated into a nonendogenous, safe harbor locus in a site-directed transgenic mouse assay. The selected genomic region corresponding to the selected enhancer element was synthesized by Twist Biosciences as double-stranded DNA. DNA fragments were subcloned into a lacZ reporter vector (Addgene no. 139098) using Gibson assembly (New England Biolabs). The final transgenic vector consisted of the predicted enhancer-promoter-reporter sequence flanked by homology arms complimentary to the H11 locus in the mouse genome. Correct assembly of the cloned constructs was confirmed using the Primordium long-read sequencing service. Transgenic mice were generated using our pronuclear injection protocol (73). Briefly, sgRNAs (50 ng/μL) targeting the H11 locus and Cas9 protein (Integrated DNA Technologies, catalog 1081058; final concentration: 20 ng/μL) were mixed in microinjection buffer (10  mM Tris, pH 7.5, 0.1 mM EDTA). The mix was injected into the pronuclei of single-cell-stage fertilized FVB/NJ (The Jackson Laboratory, strain no. 001800) embryos obtained from the oviducts of superovulated 7- to 8-week-old FVB/NJ female mice mated with 7- to 8-week-old FVB/NJ male mice. The injected embryos were cultured in M16 medium supplemented with amino acids at 37°C under 5% CO_2_ for approximately 2 hours and transferred into the oviducts of pseudopregnant CD-1 (Charles River Laboratories, strain code 022) surrogate mothers. Embryos were collected for downstream experiments at the E9.5 stage. β-Gal staining was performed in our standardized pipeline with modification. Embryos were fixed with 4% PFA for 20 minutes while rolling at room temperature. The embryos were genotyped for the integration of the transgenic construct. Embryos positive for transgene integration into the H11 locus and at the correct developmental stage were imaged on a Leica MZ16 microscope.

The enhancer sequences tested were assigned unique VISTA IDs (Figure 5): E1-WT – hs2662, E1-ΔNKX2-5/MEF2_1&ΔMEF2_2 – hs2662.1, E1-ΔNKX2-5/MEF2_1 – hs2662.2, and E1-ΔMEF2_2 – hs2662.3. Further details of these elements can be found at the VISTA Enhancer Browser (74).

### WGS analysis.

Genomic DNA extracted from LV samples from patients with unexplained DCM was sequenced using Illumina HiSeq instruments as previously described (75). Briefly, all sequencing reads were aligned to hg38 (GRCh38) using BWA-MEM with the -Y option (version 0.7.15) (76). Single nucleotide variants (SNVs) and small indels were identified using the Genome Analysis Tool Kit (GATK) (version 4.1) Haplotype Caller tool (77). The SNVs were annotated using vcfanno (version 0.3.2), dbSNP (build 151), the 1000 Genomes Project (phase 3), gnomAD (version 3.0), SnpEff (version 4.3t, annotation database GRCh38.86) (78), and dbNSFP (version 3.5a) (79). High-quality variants (passed the GATK Variant Score Quality Recalibration [VSQR] truth sensitivity threshold of 99.5 for SNVs and 99.0 for indels, a minimum depth [DP] of 10, genotype quality [GQ)] of 20 or higher, and quality [QUAL] of 30 or higher) were filtered for rare variants (defined as MAF < 2.00 × 10^–5^ in gnomAD, version 3.1).

### Statistics.

Statistical analyses were performed using an unpaired, 2-tailed *t* test, a χ^2^ test, the Wilcoxon rank-sum test, or a binomial test and corrected for multiple testing using the R statistical package (version 4.1.3) as noted in the text and figure legends. A *P* value of less than 0.05 was considered significant. All data are presented as the mean ± SD.

### Study approval.

Studies of mouse models were conducted using protocols that were reviewed and approved by the IACUC of Harvard Medical School (Boston, Massachusetts, USA). All cardiac samples were collected and processed in an anonymized manner according to the approved protocols reviewed by the ethic committee at Mazankowski Alberta Heart Institute (Edmonton, Alberta, Canada). All animal work was reviewed and approved by the Lawrence Berkeley National Laboratory Animal Welfare and Research Committee (Berkley, California, USA).

### Data availability.

The data that support the findings of this study are available within the article, the online supplemental files, including the Supporting Data Values file, and in the publicly available Gene Expression Omnibus (GEO) database (GSE283867, GSE282670, GSE282693) and the Database of Genotypes and Phenotypes (dbGaP) (accession no. phs001735; https://www.ncbi.nlm.nih.gov/projects/gap/cgi-bin/study.cgi?study_id=phs001735.v2.p1). Additional requests may be sent to the corresponding authors.

## Author contributions

YK, JGS, and CES conceived the study and designed experiments. YK, SWK, DS, MN, MS, JH Lee, OL, LKW, JH Letendre, FX, JKE, KG, PS, BG, NWD, N Singhal, AS, CNT, and JMG established genetically engineered hiPSC-CMs and analyzed them. YK, HW, N Slaven, OL, ML, and JMG carried out mouse studies. QM conducted total protein gel analysis. GYO provided human cardiac tissue samples. YK, JGS, and CES wrote the manuscript. YK, ZA, WTP, DED, LAP, AV, CSC, JGS, and CES supervised the study. The authorship order was assigned on the basis of the degree of contribution to the manuscript.

## Supplementary Material

Supplemental data

Unedited blot and gel images

Supporting data values

## Figures and Tables

**Table 1:**
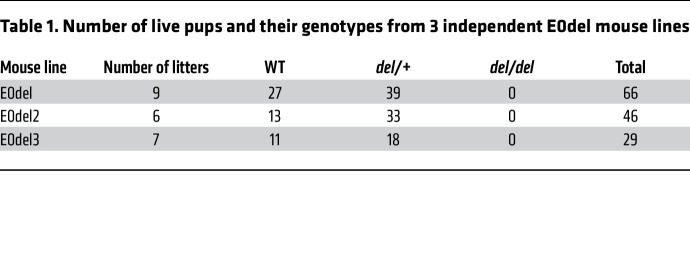
Number of live pups and their genotypes from 3 independent E0del mouse lines

**Table 2:**
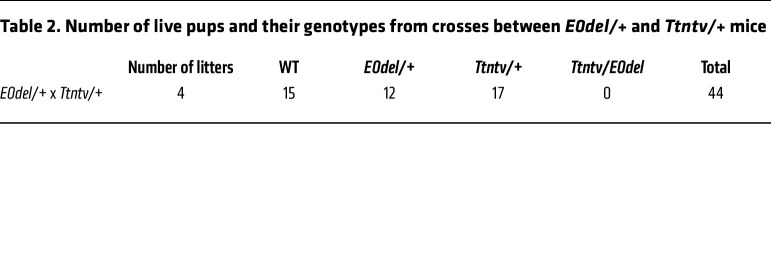
Number of live pups and their genotypes from crosses between *E0del/+* and *Ttntv/+* mice

## Version 1 12/17/2024

In-Press Preview

## Version 2 02/17/2025

Electronic publication
