# Supplementary material for: Regulation of sarcomere formation and function in the healthy heart requires a titin intronic enhancer
Source: J Clin Invest. 2024 Dec 17;135(4):e183353. doi: 10.1172/JCI183353 (PMC11827849; doi:10.1172/JCI183353)
Supplement: Supplemental data [file jci-135-183353-s083.pdf]

Supplemental Materials for:

Regulation of sarcomere formation and function in the healthy heart requires a titin intronic enhancer

Yuri Kim[1,2,^,*], Seong Won Kim[1,^], David Saul[2,^], Meraj Neyazi[1,3,^], Manuel Schmid[1,4,^], Hiroko Wakimoto[1,^], Neil Slaven[5], Joshua H. Lee[6], Olivia Layton[1], Lauren K. Wasson[1], Justin H. Letendre[6], Feng Xiao[7], Jourdan K. Ewoldt[6], Konstantinos Gkatzis[8], Peter Sommer[8], Bénédicte Gobert[8], Nicolas Wiest-Daesslé[8], Quentin McAfee[9], Nandita Singhal[2], Mingyue Lun[1], Joshua M. Gorham[1], Zolt Arany[9], Arun Sharma[10], Christopher N. Toepfer[11,12], Gavin Y. Oudit[13,14], William T. Pu[7,15], Diane E. Dickel[5,16], Len A. Pennacchio[5,17,18], Axel Visel[5,17,19], Christopher S. Chen[6], J. G. Seidman[1,#], Christine E. Seidman[1,2,20,#,*]

[1]Department of Genetics, Harvard Medical School, Boston, MA, USA

[2]Division of Cardiovascular Medicine, Brigham and Women's Hospital, Boston, MA, USA

[3]Department of Cardiology, University Heart and Vascular Center Hamburg, University Medical Center Hamburg-Eppendorf, Hamburg, Germany

[4]German Heart Center, Technical University of Munich, Munich, Germany

[5]Environmental Genomics and Systems Biology Division, Lawrence Berkeley National Laboratory, Berkeley, CA, USA

[6]Department of Biomedical Engineering, Boston University, Boston, MA, USA

[7]Department of Cardiology, Boston Children's Hospital, Harvard Medical School, Boston, MA, USA

[8]Ksilink, 67000, Strasbourg, France

[9]Cardiovascular Institute, Department of Medicine, Perelman School of Medicine, University of Pennsylvania, Philadelphia, PA, USA.

[10]Smidt Heart Institute, Cedars-Sinai Medical Center, Los Angeles, CA, USA

[11]Division of Cardiovascular Medicine, Radcliffe Department of Medicine, University of Oxford, Oxford, UK

[12]Wellcome Centre for Human Genetics, University of Oxford, Oxford, UK

[13]Department of Medicine, University of Alberta, Edmonton, Alberta, Canada

[14]Mazankowski Alberta Heart Institute, Edmonton, Alberta, Canada

[15]Harvard Stem Cell Institute, Cambridge, MA, USA

[16]Currently affiliated with Octant Inc., Emeryville, CA, USA

[17]U.S. Department of Energy Joint Genome Institute, One Cyclotron Road, Berkeley, CA, USA

[18]Comparative Biochemistry Program, University of California, Berkeley, CA, USA

[19]School of Natural Sciences, University of California, Merced, CA, USA

[20]Howard Hughes Medical Institute, Chevy Chase, MD, USA

[^,#]These authors contributed equally

*Corresponding authors

*Corresponding Authors:

Yuri Kim
Division of Cardiovascular Medicine, Brigham and Women's Hospital
75 Francis St
Boston, MA 02115
Phone: 857-307-4000
Email: ykim@genetics.med.harvard.edu

Christine E. Seidman
NRB Room 265
Department of Genetics, Harvard Medical School
77 Ave Louis Pasteur
Boston, MA 02115
Phone: 617-432-7871
Email: cseidman@genetics.med.harvard.edu

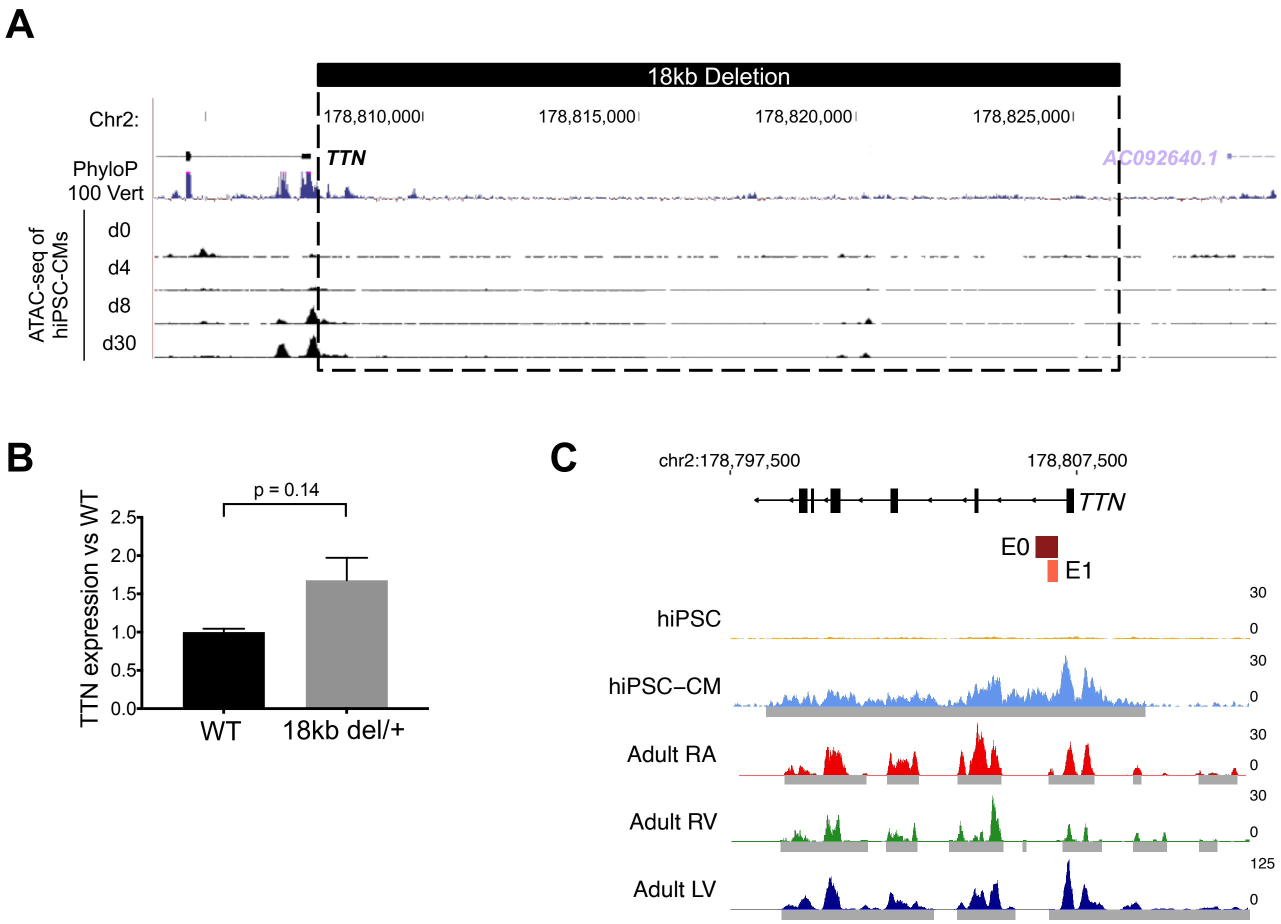

Supplemental Figure 1. TTN expression after deletion of its putative regulatory elements. (A) Genomic location of 18 kb sequences upstream of the *TTN* transcriptional start site. The sequences were deleted using CRISPR/Cas9 to investigate their role in transcriptional regulation of *TTN*. **(B)** *TTN* gene expression after deleting the 18 kb sequences upstream of its transcription start site. Error bars represent standard error of mean. n=3 per group. **(C)** H3K27ac ChIP-seq data of undifferentiated hiPSCs, hiPSC-CMs (differentiation day 30), and adult human hearts from the NIH Roadmap Epigenomics Consortium (1). TTN exons are represented as black boxes at the top, with introns depicted as black lines. The TTN enhancers E0 and E1 are highlighted by crimson and orange boxes, respectively. Normalized read counts from ChIP-seq are shown as colored graphs for hiPSCs and hiPSC-CMs. For adult human heart tissue, the -log10 Poisson p-value of ChIP-seq data, relative to expected local background counts, is displayed as colored graphs. Significant peaks are highlighted by a grey box beneath the signals. All panels are drawn on the same coordinates.

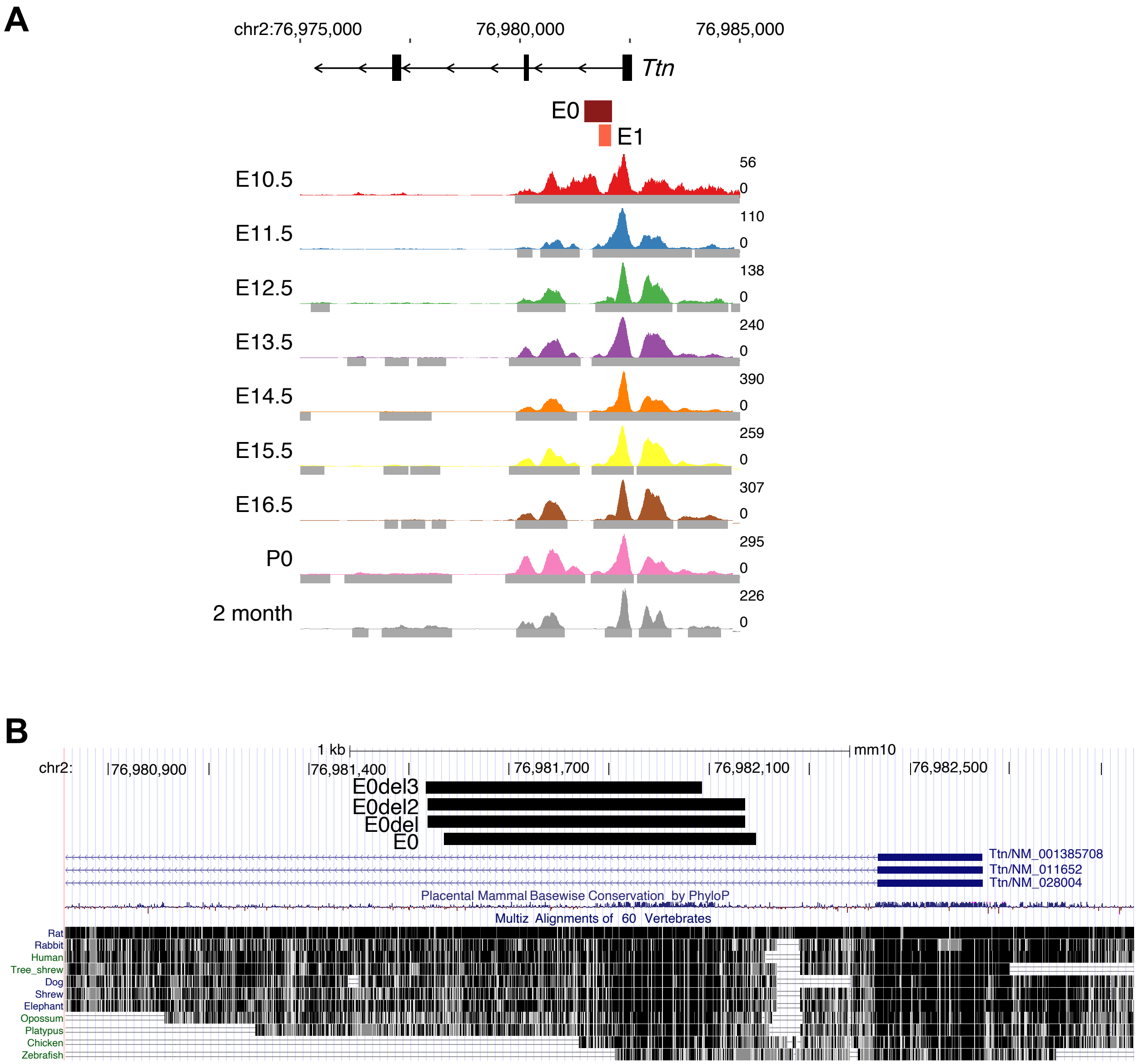

Supplemental Figure 2. (A) H3K27ac ChIP-seq data of developing mice obtained from the ENCODE Project Consortium (2). Mouse Ttn exons are shown as black boxes at the top, with introns shown as black lines. The TTN enhancers E0 and E1, lifted over from human genomic coordinates, are highlighted by crimson and orange boxes. -log10 of the Poisson p-value of ChIP-seq are shown as colored graphs. Significant peaks are shown as grey box. Developmental time points are shown on the left. **(B)** Deletion map of the three genetically engineered mouse lines carrying TTN E0 deletion (E0del, E0del2, E0del3). The most bottom black box represents the TTN E0 sequences in mice (601 bp), while the top three black lines indicate the deleted sequences in each mouse line with E0 deletion (E0del1 645 bp, E0del2 635 bp, E0del3 553 bp). The edited sequences in each mouse line were identified via MiSeq analysis. This map was adapted from the UCSC Genome Browser (3).

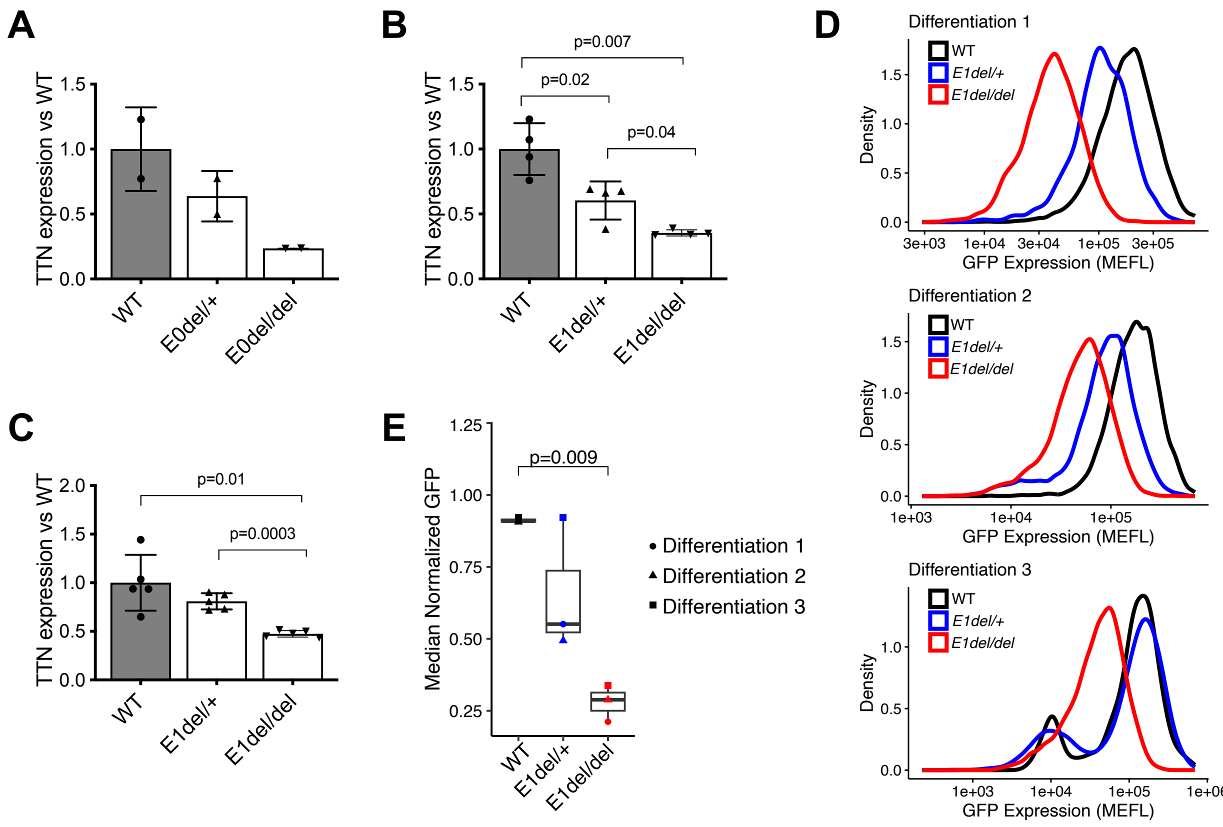

Supplemental Figure 3. TTN expression in hiPSC-CMs with TTN enhancer deletion. Pseudobulk analysis of single cell RNA-sequencing data from hiPSC-CMs at differentiation day 30 harboring E0 deletion **(A)** and E1 deletion **(B)**. TTN expression values normalized to WT are shown. Each dot represents independent differentiation. p-values were calculared using t-test with Welch's correction. **(C)** TTN expression in hiPSC-CMs with E1 deletion in WTC11 genetic background. Multiplexed single nuclei RNA-sequencing was performed using hiPSC-CMs at differentiation day 30. Data from 5 independent differentiation. P-values were calculated using t-test. **(D)** TTN-GFP quantification using flow cytometry in hiPSC-CMs with E1 deletion. Each plot represents data from independent differentiation, which demonstrated dosage-dependent TTN-GFP expression upon E1 deletion. Composite data is shown in Figure 3B. MEFL: mean equivalent fluorochrome. **(E)** Quantification of normalized GFP expression values in hiPSC-CMs with E1 deletion. Median values of normalized GFP values (normalized to WT mean value within each differentiation set) are shown. Values from three independent differentiation. P-values were calculated using t-test.

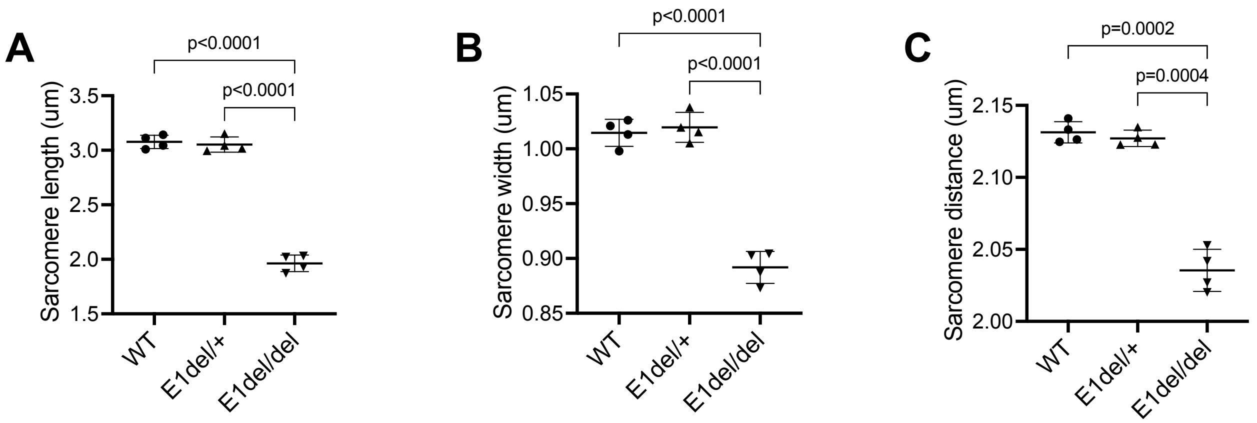

Supplemental Figure 4. Sarcomere characterization of hiPSC-CMs with E1 deletion. Characterization of sarcomeres by measurements of their length **(A)**, width **(B),** and distance **(C)**. Cardiomyocytes were collected at differentiation day 30-35 from four independent differentiation sets. Each dot represents mean values of data collected from six 96 wells. P-values were calculated using t-test with Welch's correction.

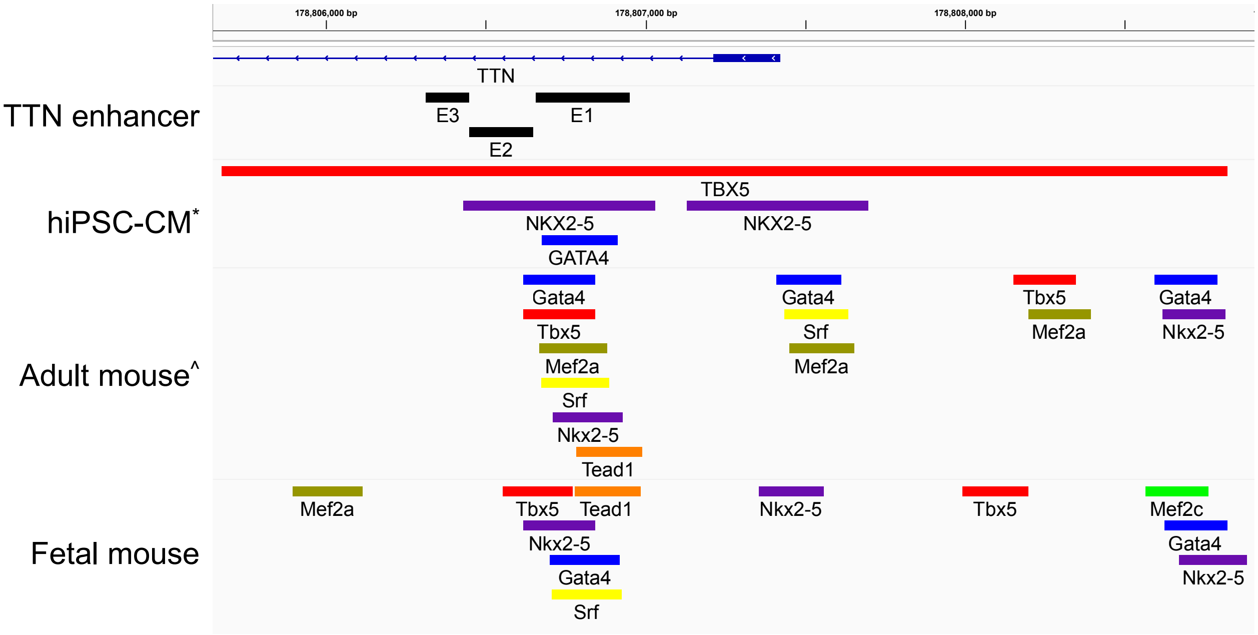

Supplemental Figure 5. Cardiac transcription factors that interact with TTN regulatory elements. Previously published ChIP-seq data (4-6) demonstrated that multiple cardiac transcription factors including NKX2-5 and MEF2 occupy DNA sequences within TTN regulatory elements in hiPSC-CMs and mouse heart. ChIP-seq data *not available for MEF2 family and ^only available for MEF2A.

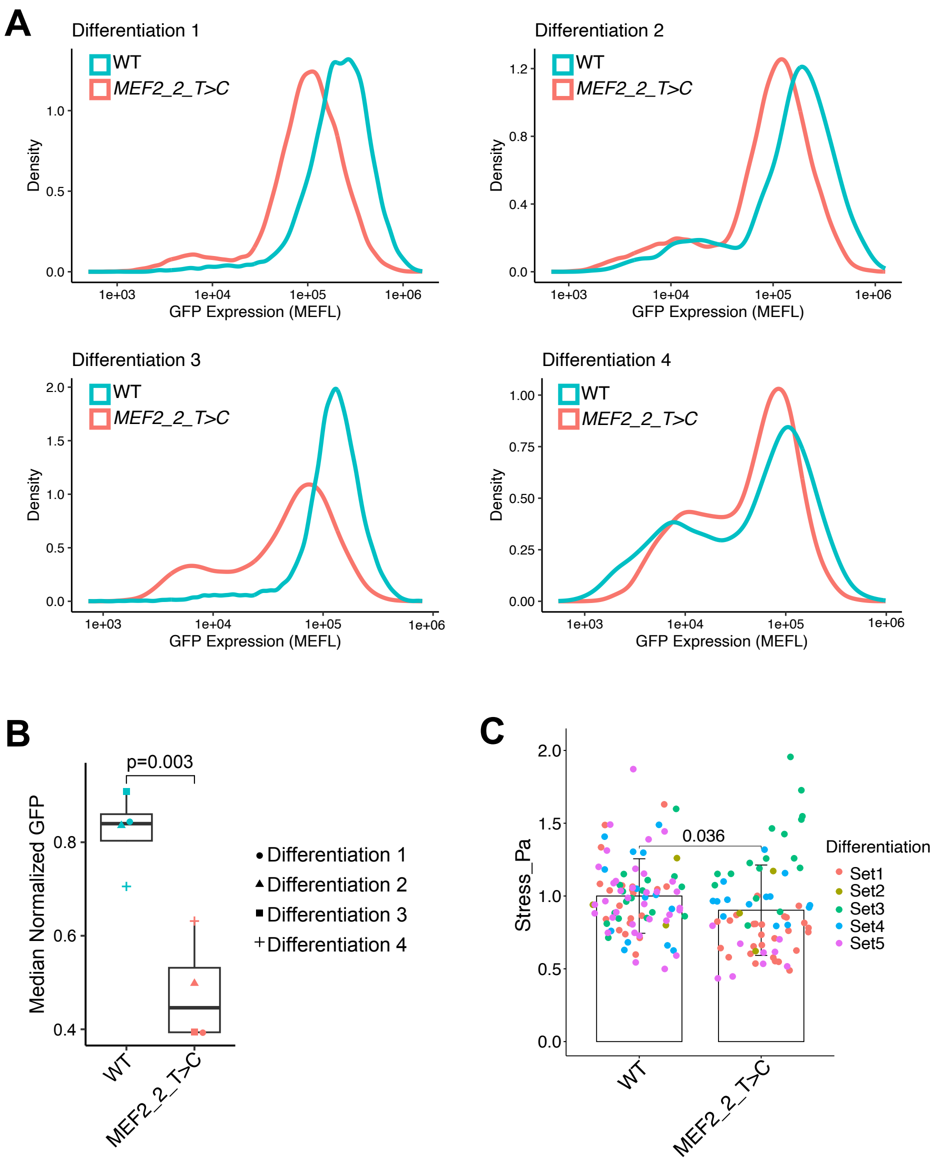

Supplemental Figure 6. TTN-GFP expression in hiPSC-CMs carrying the rare E1 variant identified in a DCM patient. (A) GRCh38/hg38 chr2:178,806,843T>C change in a conserved predicted MEF2 binding site (MEF2_2T>C) within E1 was introduced into hiPSC-CMs in a biallelic manner. Flow cytometry was used to measure and calibrate GFP intensity of the mutant hiPSC-CMs at differentiation day 30. Each panel demonstrates data from independent differentiation. MEFL: mean equivalent fluorochrome. **(B)** Quantification of normalized GFP expression. Median values of normalized GFP expression (normalized to WT mean value within each differentiation set) are shown. Total four independent differentiation. p-value was calculated using t-test. **(C)** Cardiac microtissue force measurement. Stress values were normalized to WT within each differentiation. Each dot represents one cardiac microtissue. Total five independent differentiations. P-value was calculated using t-test with Welch's correction.

Supplemental Table 1. Echocardiographic data of mice with E0 deletion

Animal ID	Sex	Genotype	Age (week)	Weight (g)	LVPWd (mm)	LVDd (mm)	FS (%)
5102	Male	*E0del/+*	34	31.6	0.81	3.71	33.2
5116	Male	*E0del/+*	29	32.3	0.82	3.76	39.2
			47	32.4	0.82	3.56	48.3
			87	34.6	0.8	4.05	35.5
5117	Male	*E0del/+*	29	31.6	0.82	3.60	45.5
			47	35.9	0.86	3.93	39.5
			87	39.9	0.87	3.26	42.2
1625	Female	*E0del/+*	65	26.3	0.76	3.44	41.0
1608	Female	*E0del/+*	65	25.4	0.76	3.44	40.5
1616	Female	*E0del/+*	65	26.6	0.81	3.23	45.4
5137	Male	*E0del/+*	41	28.9	0.72	3.65	32.5
5127	Male	*E0del2/+*	42	28.8	0.71	3.94	42.2
5128	Male	*E0del2/+*	42	35.6	0.75	4.15	38.4
5118	Male	*E0del3/+*	29	30.9	0.89	3.69	40.9
5131	Male	*E0del3/+*	22	30.3	0.78	3.93	40.5
5132	Male	*E0del3/+*	22	29.7	0.75	3.66	46.2

FS: fractional shortening, LVDd: left ventricle dimension at diastole, LVPWd: left ventricular posterior wall thickness at diastole

Supplemental Table 2A. Allele-specific expression of TTN single nucleotide polymorphisms in *E0del/+* mice with hybrid genetic background (C57BL/6 E0del/+ X 129SvEV WT)

Genomic locus (mm10)	Genotype	ID	Organ	C57BL/6 allele	129SvEv allele	B6/129SvEv ratio
chr2:76,969,682 C57BL/6 G 129SvEv A	WT	1661	LV	3972 (49%)	4060 (50%)	0.98
			SKM	7822 (48%)	8566 (52%)	0.91
		1663	LV	9320 (46%)	10886 (54%)	0.86
			SKM	8761 (46%)	10273 (54%)	0.85
	E0del/+	1660	LV	5286 (33%)	10572 (67%)	0.5
			SKM	4573 (31%)	10234 (69%)	0.45
		1662	LV	6097 (32%)	13215 (68%)	0.46
			SKM	5566 (30%)	13273 (70%)	0.42
		1665	LV	4979 (32%)	10452 (68%)	0.48
			SKM	5434 (32%)	11443 (68%)	0.47
chr2:76,969,687 C57BL/6 A 129SvEv G	WT	1661	LV	4174 (51%)	3948 (48%)	1.06
			SKM	8061 (49%)	8226 (50%)	0.98
		1663	LV	9756 (48%)	10533 (52%)	0.93
			SKM	9149 (48%)	9900 (52%)	0.92
	E0del/+	1660	LV	5465 (35%)	10299 (65%)	0.53
			SKM	4747 (32%)	9954 (68%)	0.48
		1662	LV	6404 (33%)	12903 (67%)	0.5
			SKM	5832 (31%)	12986 (69%)	0.45
		1665	LV	5209 (34%)	10208 (66%)	0.51
			SKM	5715 (34%)	11223 (66%)	0.51
chr2:76,969,699 C57BL/6 A 129SvEv G	WT	1661	LV	4570 (52%)	4153 (47%)	1.10
			SKM	8903 (51%)	8509 (49%)	1.05

		1663	LV	10930 (50%)	10803 (50%)	1.01
			SKM	10422 (51%)	10090 (49%)	1.03
	E0del/+	1660	LV	6069 (37%)	10510 (63%)	0.58
			SKM	5339 (34%)	10174 (66%)	0.52
		1662	LV	7238 (35%)	13250 (65%)	0.55
			SKM	6633 (33%)	13278 (67%)	0.5
		1665	LV	6240 (38%)	10384 (62%)	0.6
			SKM	6682 (37%)	11547 (63%)	0.58

Table S2B. Significantly decreased expression of the TTN allele carrying E0 deletion (C57BL/6) compared to the TTN allele without E0 deletion (129SvEv)

	**B6/129SvEv ratio**		**p-value**
	**WT**	***E0del/+***	
LV	0.99 ± 0.09	0.52 ± 0.05	8.36E-06
SKM	0.96 ± 0.07	0.49 ± 0.05	1.09E-06

LV: left ventricle, SKM: skeletal muscle

Supplemental Table 3. Predicted transcriptional factor binding motifs within E1

TF	Potential binding motif
AMYB	106(GCAACTGATA),119(CAGCAGTTGA)
AR-HALFSITE	88(GTGTTCTGTC),191(GTGTGCTTAA)
BMAL1	161(ACCACATG),163(CACATGCA)
BMYB	106(GCAACTGATA),119(CAGCAGTTGA)
CEBP	208(ATTGAGTAAT),208(ATTGAGTAAT)
FOXA1	260(GAAGTAAACA)
FOXH1	121(GCAGTTGATTCC)
FOXK2	261(AAGTAAACATTT)
FOXL2	260(GAAGTAAACATT)
FOXM1	260(GAAGTAAACA)
FOXO1	263(GTAAACAT)
FOXO3	262(AGTAAACA)
GATA3	171(TTTTATCA),201(CTTTATCA)
GATA4	171(TTTTATCAGA)
GATA6	171(TTTTATCAGA)
HOXD13	36(GCTCTTAAAA),130(TCCCTTAAAA)
ISL1	130(TCCCTTAA)
KLF3	144(ATCAGGGTGTAGCTG)
KLF5	147(AGGGTGTAGC)
MAFA	114(TACCTCAGCA)
MEF2A	182(CTATTCTTGG)
MEF2B	38(TCTTAAAATAAA),181(GCTATTCTTGGT)
MEF2C	38(TCTTAAAATAAA),181(GCTATTCTTGGT)
MEIS1	72(GACTGACAGC),73(ACTGACAGCA),91(TTCTGTCACT)
MYB	107(CAACTGAT),120(AGCAGTTG)
N-MYC	160(TACCACATGC)
NANOG	124(GTTGATTCCC),140(AGTCATCAGG)
NKX2.1	35(TGCTCTTAAA),58(AACTCTCCAA)
NKX2.2	35(TGCTCTTAAA)
NKX2.5	34(ATGCTCTTAA),57(AAACTCTCCA)
NPAS	161(ACCACATG),163(CACATGCA)
NR5A2	219(TAACCTTGAA)
NR5A2	219(TAACCTTGAA)
OLIG2	76(GACAGCAGGC)
PAX5	62(CTCCAAGGTTGACTGA)
PAX8	63(TCCAAGGTTGACTGA)
PBX1	71(TGACTGACAGCA)

PBX3	70(TTGACTGACAGC)
PKNOX1	70(TTGACTGACAGC)
SCL	76(GACAGCAG),78(CAGCAGGC),119(CAGCAGTT)
TATA-BOX	130(TCCCTTAAAAAG),132(CCTTAAAAAGTC),250(GTCTAGAAAAGA)
TGIF1	73(ACTGACAG),74(CTGACAGC),93(CTGTCACT)
TGIF2	27(AAATGTCA),72(GACTGACA),75(TGACAGCA)

Each row represents transcription factor (TF) with predicted binding motifs, DNA sequences and base pair position within E1.

Supplemental Table 4. gRNA and HDR template sequences used for genetic engineering of hiPSCs

Sequence Name	gRNA Sequence
Mouse E0del gRNA1	5'-AATTTTTTTTGAAGATACAC
Mouse E0del gRNA2	5'-GTATGGGATGTCCTAGAACC
Human E0del gRNA1	5'-CTTAATATTCTAAAAGTTG
Human E0del gRNA2	5'-TAGGTATTCTATGTCTAAG
Human E1del gRNA1	5'-CTTAATATTCTAAAAGTTG
Human E1del gRNA2	5'-TTCAGCCCCAGTTAGACAA
Human MEF2_2T>C gRNA	5'-CATTTTATCAGAGCTATTCT
Human MEF2_2T>C HDR template	5'-GTGTAGCTGATACCACATGCATTTTATCAGAGCTATTCCTGG TGTGCTTAACTTTATCATTGAGTAATATAACCTTGAACATTCTC AACAGGCAAAGAGAGTCTAGAAAAGAAGTAAACATTTCTCT

Supplemental Table 5. TTN enhancer construct sequences used for mouse transgenic enhancer assays

Transgenic construct name	Sequence
WT (E1)	5’-TGAGGTCTGAAATGTTGAGAGAAATGTTTACTTCTTTTCTAGACTCTCTTTGCCTGTTGAGAATGTTCAAGGTTATATTACTCAATGATAAAGTTAAGCACACCAAGAATAGCTCTGATAAAATGCATGTGGTATCAGCTACACCCTGATGACTTTTTAAGGGAATCAACTGCTGAGGTATCAGTTGCAGGACAGTGACAGAACACTTGCCTGCTGTCAGTCAACCTTGGAGAGTTTAGGAGAGTTTATTTTAAGAGCATGACATTTTAGACGCATACCTTCAGCCCCAGTTAGA
∆NKX2-5/ MEF2_1	5’-TTGAGGTCTGAAATGTTGAGAGAAATGTTTACTTCTTTTCTAGACTCTCTTTGCCTGTTGAGAATGTTCAAGGTTATATTACTCAATGATAAAGTTAAGCACACCAAGAATAGCTCTGATAAAATGCATGTGGTATCAGCTACACCCTGATGACTTTTTAAGGGAATCAACTGCTGAGGTATCAGTTGCAGGACAGTGACAGAACACTTGCCTGCTGTCAGTCAACCTTGGAGAGTTTAGGAGAGGACATTTTAGACGCATACCTTCAGCCCCAGTTAGA
∆MEF2_2	5’-TTGAGGTCTGAAATGTTGAGAGAAATGTTTACTTCTTTTCTAGACTCTCTTTGCCTGTTGAGAATGTTCAAGGTTATATTACTCAATGATAAAGTTAAGCACACTCTGATAAAATGCATGTGGTATCAGCTACACCCTGATGACTTTTTAAGGGAATCAACTGCTGAGGTATCAGTTGCAGGACAGTGACAGAACACTTGCCTGCTGTCAGTCAACCTTGGAGAGTTTAGGAGAGTTTATTTTAAGAGCATGACATTTTAGACGCATACCTTCAGCCCCAGTTAGA
∆NKX2-5/ MEF2_1& ∆MEF2_2	TTGAGGTCTGAAATGTTGAGAGAAATGTTTACTTCTTTTCTAGACTCTCTTTGCCTGTTGAGAATGTTCAAGGTTATATTACTCAATGATAAAGTTAAGCACACTCTGATAAAATGCATGTGGTATCAGCTACACCCTGATGACTTTTTAAGGGAATCAACTGCTGAGGTATCAGTTGCAGGACAGTGACAGAACACTTGCCTGCTGTCAGTCAACCTTGGAGAGTTTAGGAGAGGACATTTTAGACGCATACCTTCAGCCCCAGTTAG

Supplemental References

1. Kundaje A, et al. Integrative analysis of 111 reference human epigenomes. *Nature*. 2015;518(7539):317–330.

2. Moore JE, et al. Expanded encyclopaedias of DNA elements in the human and mouse genomes. *Nature*. 2020;583(7818):699–710.

3. Haeussler M, et al. The UCSC Genome Browser database: 2019 update. *Nucleic Acids Res*. 2019;47(D1):D853–D858.

4. Akerberg BN, et al. A reference map of murine cardiac transcription factor chromatin occupancy identifies dynamic and conserved enhancers. *Nat Commun*. 2019;10(1):4907.

5. Ang Y-S, et al. Disease Model of GATA4 Mutation Reveals Transcription Factor Cooperativity in Human Cardiogenesis. *Cell*. 2016;167(7):1734-1749.e22.

6. Benaglio P, et al. Allele-specific NKX2-5 binding underlies multiple genetic associations with human electrocardiographic traits. *Nat Genet*. 2019;51(10):1506–1517.
