# Supplementary figures and images for: Regulation of sarcomere formation and function in the healthy heart requires a titin intronic enhancer
Source: J Clin Invest. 2024 Dec 17;135(4):e183353. doi: 10.1172/JCI183353 (PMC11827849; doi:10.1172/JCI183353)

Lanes used in the manuscript

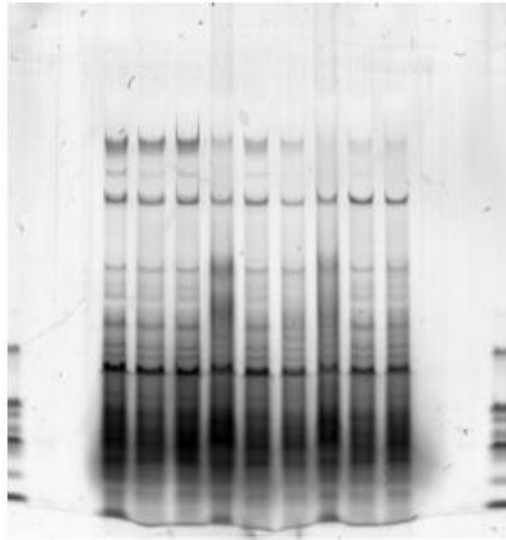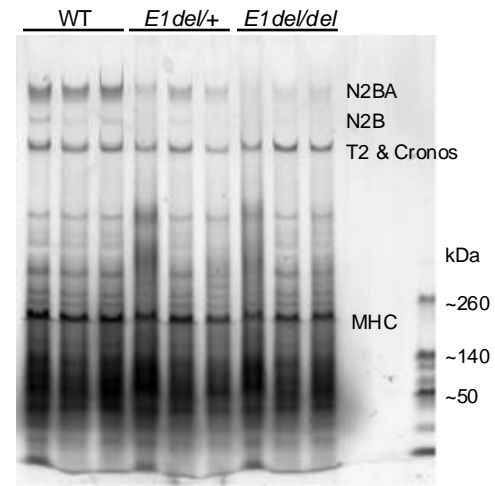

Supplement: Unedited blot and gel images [file jci-135-183353-s084.pdf]
